# PROX1 is an early driver of lineage plasticity in prostate cancer

**DOI:** 10.1172/JCI187490

**Published:** 2025-06-02

**Authors:** Zhi Duan, Mingchen Shi, Anbarasu Kumaraswamy, Dong Lin, Dhruv Khokhani, Yong Wang, Chao Zhang, Diana Flores, Eva Rodansky, Olivia A. Swaim, William K. Storck, Hannah N. Beck, Radhika A. Patel, Erolcan Sayar, Brian P. Hanratty, Hui Xue, Xin Dong, Zoe R. Maylin, Rensheng Wan, David A. Quigley, Martin Sjöström, Ya-Mei Hu, Faming Zhao, Zheng Xia, Siyuan Cheng, Xiuping Yu, Felix Y. Feng, Li Zhang, Rahul Aggarwal, Eric J. Small, Visweswaran Ravikumar, Arvind Rao, Karan Bedi, John K. Lee, Colm Morrissey, Ilsa Coleman, Peter S. Nelson, Eva Corey, Aaron M. Udager, Ryan J. Rebernick, Marcin P. Cieslik, Arul M. Chinnaiyan, Joel A. Yates, Michael C. Haffner, Yuzhuo Wang, Joshi J. Alumkal

**Affiliations:** 1 Department of Internal Medicine and; 2 Rogel Cancer Center, University of Michigan, Ann Arbor, Michigan, USA.; 3 Vancouver Prostate Centre, Vancouver, British Columbia, Canada.; 4 Department of Urologic Sciences, Faculty of Medicine, University of British Columbia, Vancouver, British Columbia, Canada.; 5 BC Cancer Research Institute, BC Cancer, Vancouver, British Columbia, Canada.; 6 Division of Human Biology, Fred Hutchinson Cancer Research Center, Seattle, Washington, USA.; 7 Helen Diller Family Comprehensive Cancer Center,; 8 Department of Radiation Oncology,; 9 Department of Epidemiology & Biostatistics, and; 10 Department of Urology, UCSF, San Francisco, California, USA.; 11 Division of Oncology, Department of Clinical Sciences, Lund, Faculty of Medicine, Lund University, Lund, Sweden.; 12 Department of Haematology, Oncology and Radiation Physics, Skåne University Hospital, Lund, Sweden.; 13 Knight Cancer Institute and; 14 Department of Biomedical Engineering, Oregon Health & Science University, Portland, Oregon, USA.; 15 Department of Biochemistry and Molecular Biology, LSU Health Shreveport, Shreveport, Louisiana, USA.; 16 Division of Hematology and Oncology, Department of Medicine, UCSF, San Francisco, California, USA.; 17 Department of Computational Medicine & Bioinformatics,; 18 Department of Biomedical Engineering,; 19 Department of Radiation Oncology, and; 20 Department of Biostatistics, School of Public Health, University of Michigan, Ann Arbor, Michigan, USA.; 21 Department of Medicine and the Institute for Urologic Oncology, David Geffen School of Medicine, UCLA, Los Angeles, California, USA.; 22 Department of Urology, University of Washington, Seattle, Washington, USA.; 23 Division of Clinical Research, Fred Hutchinson Cancer Research Center, Seattle, Washington, USA.; 24 Department of Pathology,; 25 Michigan Center for Translational Pathology, and; 26 Howard Hughes Medical Institute, University of Michigan, Ann Arbor, Michigan, USA.; 27 Department of Laboratory Medicine and Pathology, University of Washington, Seattle, Washington, USA.

**Keywords:** Cell biology, Oncology, Epigenetics, Prostate cancer

## Abstract

Lineage plasticity is recognized as a critical determinant of lethality and resistance to AR pathway inhibitors in prostate cancer. Lineage plasticity is a continuum, ranging from AR activity-low tumors, AR-null tumors that do not express a neuroendocrine prostate cancer (NEPC) program (i.e., double-negative prostate cancer [DNPC]), and AR-null NEPC tumors. Factors upregulated early in lineage plasticity are not well-characterized. The clarification of such factors is essential to identify tumors undergoing lineage plasticity or at risk of this occurring. Our integrative analysis of metastatic prostate cancer patient tumors, patient-derived xenografts, and cell models determined that PROX1 is upregulated early in the lineage plasticity continuum and progressively increases as tumors lose AR activity. We determined DNA methylation is a key regulator of PROX1 expression. PROX1 suppression in DNPC and NEPC reduces cell survival and impacts apoptosis and differentiation, demonstrating PROX1’s functional importance. PROX1 is not directly targetable with standard drug development approaches. However, affinity immunopurification demonstrated histone deacetylases (HDACs) are among the top PROX1-interacting proteins; HDAC inhibition depletes PROX1 and recapitulates PROX1 suppression in DNPC and NEPC. Altogether, our results suggest PROX1 promotes the emergence of lineage plasticity, and HDAC inhibition is a promising approach to treat tumors across the lineage plasticity continuum.

## Abstract 2

PROX1 is upregulated in prostate cancers undergoing lineage plasticity (differentiation change). HDAC inhibition blocks PROX1 and survival of prostate tumors that have undergone lineage plasticity.

## Graphical abstract

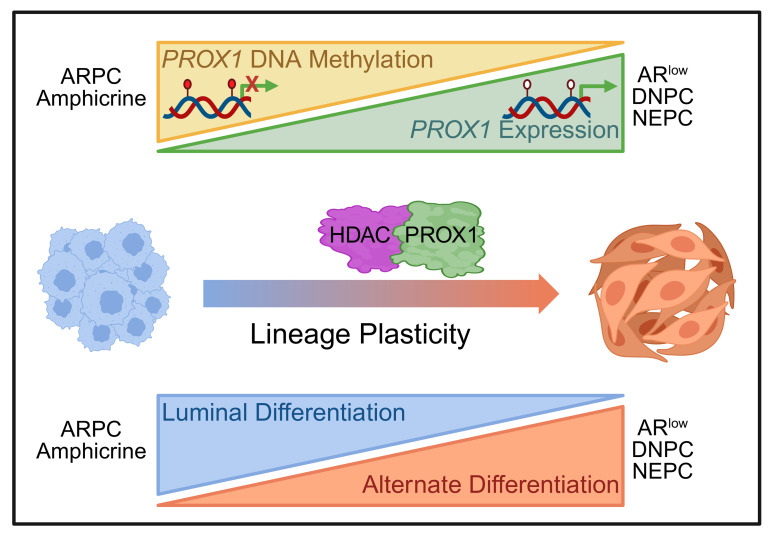

## Introduction

Prostate cancer is the second leading cause of cancer-related death in men in the United States (1), and nearly all of these deaths are due to metastatic disease. Interfering with production of male hormones that activate the androgen receptor (AR), a nuclear hormone receptor that promotes proliferation and luminal differentiation, and interfering with binding of androgens to the AR are the principal treatment strategies for metastatic prostate cancer (2). Most patients with metastatic prostate cancer respond to these therapies. However, disease progression is nearly universal. The majority of prostate cancers progressing despite AR inhibition retain expression of the AR and AR-activated pathways (3, 4). However, a subset of tumors undergoes lineage plasticity (5), losing the canonical AR-dependent program (3, 4, 6–8). These lineage plasticity tumors are particularly virulent (4, 8), and effective treatments are lacking.

Lineage plasticity, or differentiation change, in prostate cancer is most commonly exemplified by AR pathway loss and switch from a luminal to an alternate differentiation program (5). Lineage plasticity is now recognized as a critical determinant of lethality, occurring in nearly 50% of prostate cancer patients in rapid-autopsy studies (3, 9). Most efforts in the field are focused on factors that promote terminal differentiation of specific lineage plasticity subtypes such as neuroendocrine prostate cancer (NEPC). However, transcriptional profiling from our group and others demonstrates that lineage plasticity is a continuum, including AR activity–low tumors with persistent AR expression but low AR signaling, double-negative prostate cancers (DNPCs) that lose AR expression without neuroendocrine differentiation, and NEPCs that lose AR expression and undergo neuroendocrine differentiation (4, 8, 9). Factors influencing prostate cancer lineage plasticity in its incipient stages are only now beginning to be characterized (10–12). The identification of such factors is essential because there are currently no effective therapies once prostate tumors undergo lineage plasticity.

By examining prostate cancer biopsies from patients treated with the AR pathway inhibitor (ARPI) enzalutamide, we previously found that a subset of tumors underwent lineage plasticity from AR-driven prostate cancer (ARPC) to DNPC (6). *Prospero homeobox 1* (*PROX1*) was the most significantly upregulated gene in these DNPC tumors. PROX1 is a developmental transcription factor and an early neural progenitor cell driver that regulates stemness and cell fate (13–18). PROX1 is linked to the aggressiveness of several epithelial cancers and neuroendocrine differentiation of small cell lung cancer (19, 20). However, there was limited information on PROX1 expression and its role in prostate cancer (21).

We determined that *PROX1* is upregulated early in the lineage plasticity continuum in AR activity–low tumors; *PROX1* progressively increases in DNPC and is higher still in NEPC. We determined that *PROX1* is epigenetically regulated through DNA methylation. *PROX1* overexpression experiments led to reduced *AR* expression but very little change in NEPC markers, suggesting that *PROX1* upregulation alone is insufficient to promote NEPC lineage plasticity. On the other hand, *PROX1* RNA interference experiments in DNPC and NEPC established a key role of PROX1 in regulation of important cancer hallmarks that sustain lineage plasticity cells. Finally, we determined that histone deacetylase (HDAC) proteins are among the top PROX1-interacting proteins, and HDAC inhibition blocks PROX1 expression and suppresses survival of DNPC and NEPC cells. In summary, PROX1 activation occurs early in castration-induced adaptation or transdifferentiation and contributes to aggressive phenotypes, including DNPC and NEPC. HDAC inhibition is a promising approach to block PROX1 and impair the survival of DNPC and NEPC tumors.

## Results

### PROX1 is upregulated in patient samples exhibiting AR pathway loss and lineage plasticity.

**Figure 1:**
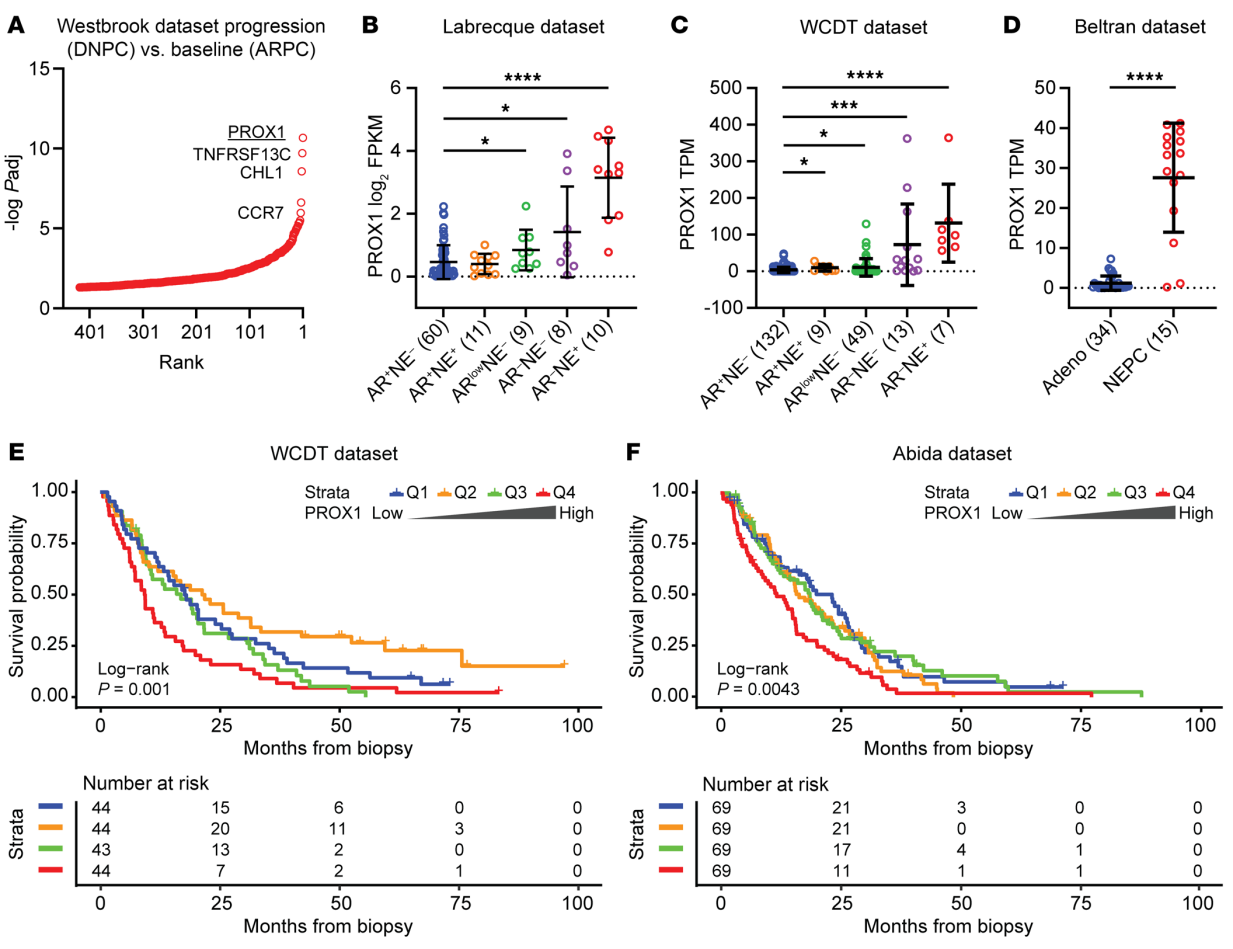
PROX1 is upregulated in patient samples exhibiting AR pathway loss and lineage plasticity. (**A**) Differentially upregulated genes ranked by adjusted *P* (*P*adj) value in prostate cancer patient tumors that converted to DNPC after enzalutamide treatment are shown from the Westbrook et al. 2022 cohort (6). *PROX1* is the top-ranked gene. (**B**–**D**) *PROX1* mRNA levels were quantified by RNA-Seq in the indicated molecular subtypes of prostate cancer patient tumors from 3 different cohorts: Labrecque et al. 2019 (*n* = 98) (9) (**B**), WCDT (*n* = 210) (8, 22) (**C**), and Beltran et al. 2016 (*n* = 49) (7) (**D**). Molecular subtypes ARPC (AR^+^NE^–^), amphicrine (AR^+^NE^+^), AR activity–low (AR^low^NE^–^), DNPC (AR^–^NE^–^), and NEPC (AR^–^NE^+^) are indicated with the sample sizes of each group. Data are reported as the mean ± SD. *P* values were calculated by unpaired 2-sample Wilcoxon’s test with Benjamini-Hochberg correction for multiple comparison (**B** and **C**) and unpaired 2-sample Wilcoxon’s test (**D**). *P < 0.05; ***P < 0.001; ****P < 0.0001. (**E** and **F**) Kaplan-Meier curves represent overall survival probability for patients in the WCDT (8, 22) (**E**) or Abida et al. 2019 (23) (**F**) cohort stratified by quantiles of *PROX1* expression. Q1 represents the lowest-quartile group, and Q4 represents the highest-quartile group. The log-rank test was used to determine significance.

Medical castration is the principal treatment for metastatic prostate cancer, but progression to castration-resistant prostate cancer (CRPC) is nearly universal (2). We previously reported a series of matched, metastatic CRPC patient biopsies taken before and after treatment with the ARPI enzalutamide — one of the main treatments for CRPC tumors (6). Fifteen percent of tumors underwent lineage plasticity to DNPC, providing the first, direct clinical evidence of AR inhibitor–induced lineage plasticity and its frequency (6). In analyzing differentially expressed genes between matched DNPC tumors and baseline ARPC tumors, *PROX1* was the most significantly upregulated gene in DNPC samples (Figure 1A and Supplemental Figure 1A; supplemental material available online with this article; https://doi.org/10.1172/JCI187490DS1).

To determine the importance of PROX1 across the lineage plasticity continuum, we evaluated *PROX1* mRNA expression in additional datasets (7–9, 22). First, we examined *PROX1* expression in the Labrecque et al. dataset (9) of rapid-autopsy samples. *PROX1* was upregulated even earlier in the lineage plasticity continuum in AR activity–low tumors, and *PROX1* expression progressively increased in DNPC and NEPC (Figure 1B). We also examined the West Coast Dream Team (WCDT) dataset (8, 22) and found that *PROX1* was highest in DNPC and NEPC (Figure 1C). Using the Beltran et al. dataset (7), we determined that *PROX1* was more highly expressed in NEPC versus adenocarcinoma tumors (Figure 1D). To confirm that PROX1 protein was also upregulated, we stained metastatic CRPC patient biopsies representing the continuum of lineage plasticity. Matching our RNA-Seq results (Supplemental Figure 1B), PROX1 expression was increased in populations of cells in an AR activity–low tumor and was highly expressed in DNPC and NEPC tumors but not in ARPC (Supplemental Figure 1C). Importantly, the pre–AR inhibitor treatment ARPC tumor (2623-1) and post–AR inhibitor treatment DNPC tumor (2623-2) (Supplemental Figure 1C) were taken from the same patient, demonstrating that preexisting PROX1-high cells were not present in the pretreatment ARPC tumor we examined. Finally, we sought to determine the association between *PROX1* upregulation and patient outcomes. *PROX1* upregulation in the WCDT dataset (8, 22) (Figure 1E) and the Abida et al. dataset (23) (Figure 1F) of CRPC patient tumors was strongly associated with poor survival.

### PROX1 is upregulated in patient-derived cell lines and xenografts exhibiting AR pathway loss and lineage plasticity.

**Figure 2:**
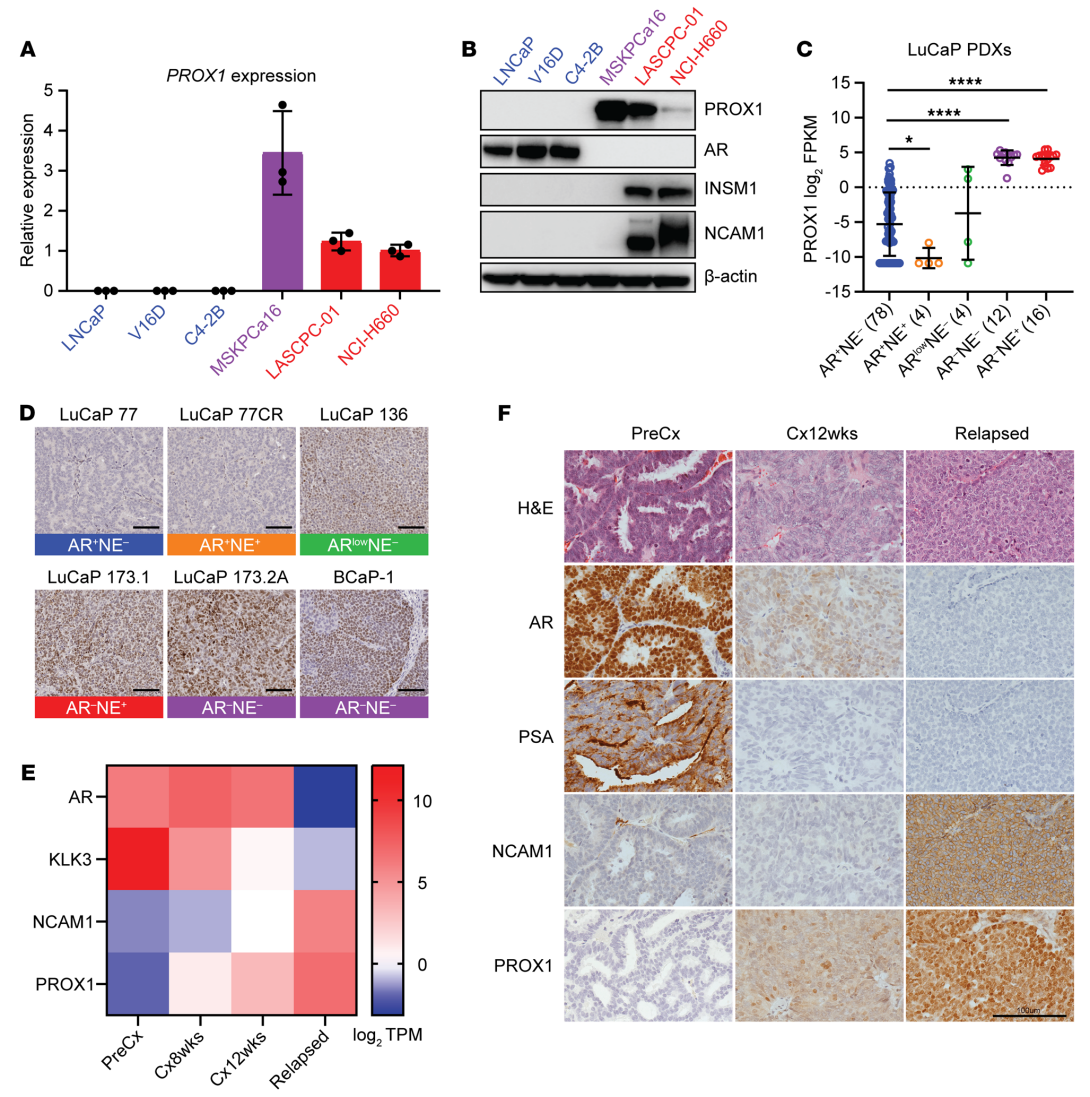
PROX1 is upregulated in patient-derived cell lines and xenografts exhibiting AR pathway loss and lineage plasticity. (**A** and **B**) *PROX1* mRNA expression was measured in the indicated prostate cancer models using reverse transcriptase quantitative PCR (RT-qPCR). β-Actin served as endogenous housekeeping control. Data are reported as the mean ± SD (*n* = 3) (**A**). PROX1 protein expression was measured in the indicated prostate cancer models using Western blotting. AR and PSA served as markers of ARPC. INSM1 served as marker for NEPC. β-Actin served as loading control (**B**). ARPC models are marked in blue, DNPC model in purple, and NEPC models in red text. (**C**) *PROX1* mRNA levels were quantified by RNA-Seq in prostate cancer patient-derived xenografts (PDXs) (*n* = 114) of the indicated molecular subtypes with their sample sizes (GEO series GSE199596). Data are reported as the mean ± SD. Statistical significance was calculated by unpaired 2-sample Wilcoxon’s test with Benjamini-Hochberg correction for multiple comparison. *P < 0.05; ****P < 0.0001. (**D**) PROX1 expression in prostate cancer PDXs was determined by IHC, and representative images with their molecular subtype are shown. Scale bars: 100 μm. (**E**) Expression levels of indicated mRNAs were quantified by RNA-Seq in LTL331 PDXs at different time points during progression from LTL331 (PreCx) to LTL331R (Relapsed). Log_2_ transcripts per million (TPM) values are indicated in the heatmap. (**F**) LTL331 progression model tumors were stained by IHC with indicated antibodies before castration (PreCx/LT331), 12 weeks after castration (Cx12wks), or after relapse (Relapsed/LT331R), and representative images are shown. Scale bar: 100 μm.

We sought to determine the PROX1 expression pattern in representative prostate cancer models, including those recapitulating the spectrum of lineage plasticity. First, we examined PROX1 expression in cell models. *PROX1* mRNA (Figure 2A) and protein (Figure 2B) were only expressed in the two NEPC cell lines — NCI-H660 and LASCPC-01, which express the NEPC markers INSM1 and NCAM1 — and a DNPC organoid, MSKPCa16, but not in ARPC cell lines.

Next, we sought to examine PROX1 expression in patient-derived xenografts (PDXs). *PROX1* mRNA was highly expressed in DNPC and NEPC PDXs but not in ARPC or amphicrine PDXs, matching the pattern in patient tumors and cell models (Figure 2C, Supplemental Figure 2A, and Supplemental Table 1). We also confirmed PROX1 protein upregulation in DNPC and NEPC PDXs using immunohistochemistry (IHC) and Western blots (Figure 2D, Supplemental Figure 2, B and C, and Supplemental Table 1). We determined that the LuCaP 136 PDX expresses the AR though prostate-specific antigen (PSA) was absent, suggesting that this is an AR activity–low model; importantly, PROX1 was also upregulated in this model (Figure 2D and Supplemental Figure 2D). These results further suggest that PROX1 is upregulated as tumors lose AR-dependence.

Given the established role of lineage plasticity in the progression of prostate cancer and the loss of AR signaling pathway during NEPC development, we hypothesized that PROX1 plays a key role even prior to NEPC differentiation change. Therefore, we utilized the LTL331/331R PDX model, which faithfully recapitulates transdifferentiation from an AR-positive adenocarcinoma to castration-resistant NEPC (24, 25). We performed IHC to measure the expression of PROX1 along with NCAM1, AR, and the AR target, PSA, using tissue sections from samples collected at multiple time points (i.e., pre-castration adenocarcinoma [LTL331], 12 weeks post-castration, and relapsed NEPC [LTL331R]). Consistent with time-course bulk RNA-Seq data (Figure 2E), PROX1 expression was not detectable in the baseline LTL331 tumors before castration (Figure 2F). However, PROX1 protein was upregulated in a subpopulation of cells in the 12-weeks-post-castration LTL331 tumors (Figure 2F). Notably, the PROX1-expressing cells at 12 weeks after castration were negative for the NEPC marker NCAM1, indicating that PROX1 upregulation occurs prior to NEPC transdifferentiation. Taken together, these results suggest that PROX1 upregulation is an early molecular event in castration-induced lineage plasticity prior to NEPC differentiation and may serve as a critical mediator of progression from adenocarcinoma to NEPC.

### DNA methylation regulates PROX1 expression.

**Figure 3:**
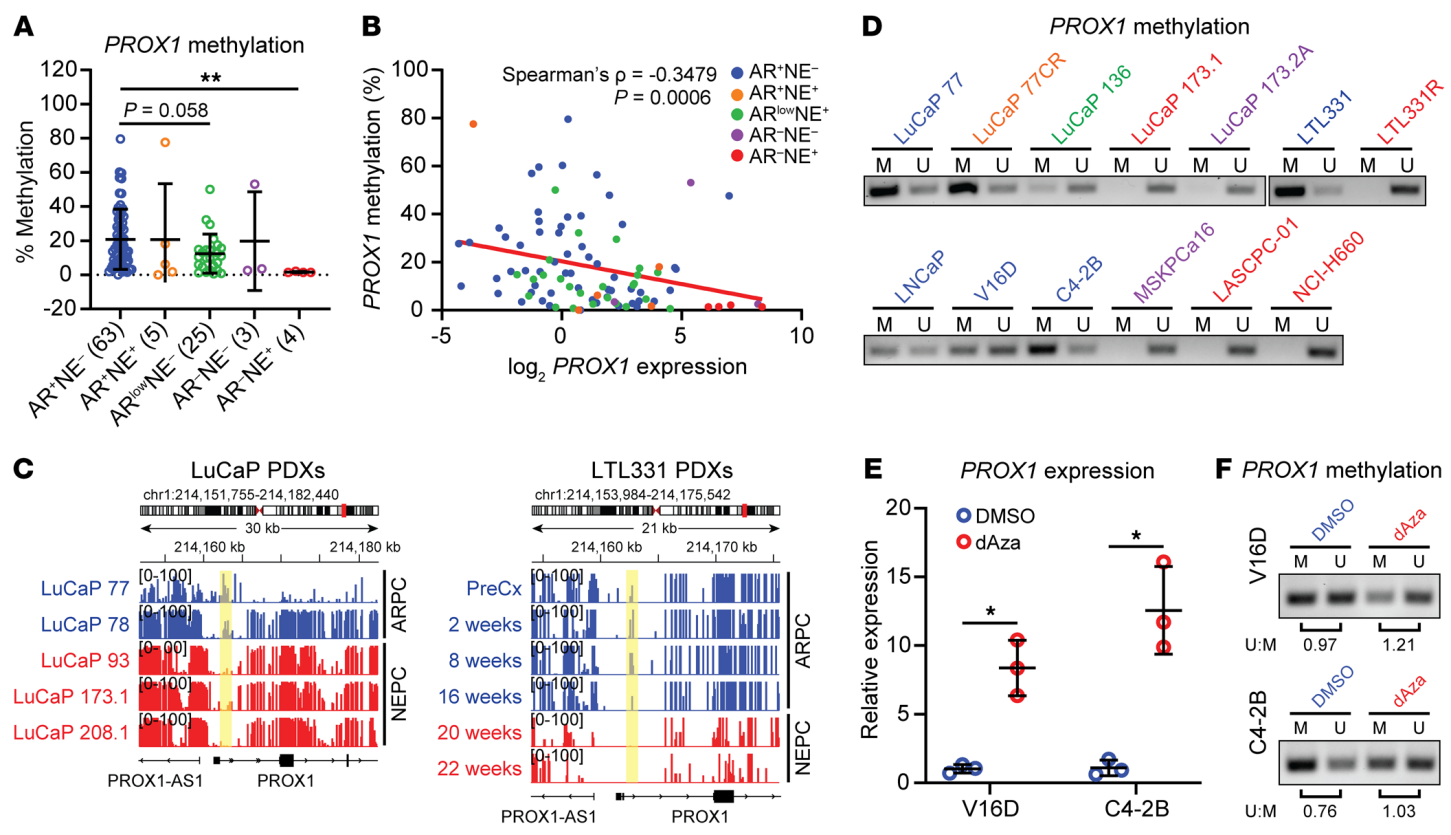
PROX1 is epigenetically regulated by DNA methylation. (**A**) *PROX1* promoter methylation in prostate cancer patient tumors was extracted from the Zhao et al. 2020 dataset (*n* = 100) (26), and 5-methylcytosine (5-mC) score is shown. Data are reported as the mean ± SD. *PROX1* promoter is significantly hypermethylated in NEPC (AR^–^NE^+^) tumors as indicated by *P* values calculated by unpaired 2-sample Wilcoxon’s test with Benjamini-Hochberg correction for multiple comparison. **P < 0.01. (**B**) Scatterplots and linear fitted lines of *PROX1* promoter DNA methylation versus log_2_ *PROX1* expression in samples from the WCDT dataset (22, 26). Spearman’s correlation coefficient (ρ) and *P* values are shown. (**C**) Genome tracks from whole-genome bisulfite sequencing analysis of indicated PDX samples indicate hypermethylation of *PROX1* promoter region (highlighted in yellow) in adenocarcinoma PDXs (blue) and hypomethylation in NEPC PDXs (red). (**D**) Methylation-specific PCR (MSPCR) was used to amplify a region of the *PROX1* promoter from prostate cancer PDXs and cell models. Methylated (M) and unmethylated (U) specific bands are shown for the indicated samples, which are color-coded: ARPC by blue, amphicrine by orange, AR activity–low by green, DNPC by purple, and NEPC by red. (**E**) The indicated cell lines were treated with 400 nM dAza (decitabine) daily for 5 days. RT-qPCR was performed to quantify *PROX1* expression with β-actin used as an endogenous control. Data are reported as the mean ± SD (*n* = 3). Statistical significance was calculated with a Student’s *t* test with Welch’s correction. *P < 0.05. (**F**) MSPCR was performed using DNA extracted from cells treated in **E**. Ratio of unmethylated (U) to methylated (M) products from densitometry analysis is shown below the respective bands.

We next sought to understand how *PROX1* is upregulated in lineage plasticity tumors. DNA methylation is a major mode of gene regulation, and tumors exhibit widespread changes in DNA methylation as they undergo lineage plasticity (7). Therefore, we examined whole-genome bisulfite sequencing (WGBS) data at the *PROX1* gene locus in patient tumor samples described in Zhao et al. (26). Importantly, these tumors also underwent RNA-Seq, enabling us to examine *PROX1* expression in specific prostate cancer subtypes defined by the Labrecque lineage plasticity subtype classifier (8). *PROX1* promoter methylation was markedly reduced in AR activity–low and significantly decreased in NEPC tumors (Figure 3A). There was a strong trend of decreased *PROX1* promoter DNA methylation in DNPC tumors as well, with two of three samples showing greatly decreased DNA methylation (Figure 3A). In examining the correlation between *PROX1* promoter DNA methylation and *PROX1* expression, we found a statistically significant inverse correlation (Figure 3B). Conversely, examination of the Sjöström et al. dataset (27) revealed that 5-hydroxymethylation — a mark that coincides with DNA demethylation (28) — was enriched at *PROX1* in NEPC tumors (Supplemental Figure 3A). There was a strong direct correlation between *PROX1* expression and 5-hydroxymethylation at the *PROX1* gene (Supplemental Figure 3B). These data demonstrate the importance of epigenetic regulation of *PROX1* through DNA methylation modifications. Importantly, many of the *PROX1* expression–high but *PROX1* DNA methylation–low/5-hydroxymethylation–high tumors belonged to subtypes with low AR activity (i.e., AR activity–low, DNPC, and NEPC) (Figure 3B and Supplemental Figure 3B). Using this same CRPC patient dataset, we found that *PROX1* expression correlated with expression of the *ten-eleven translocation 1* (*TET1*) DNA demethylase, which catalyzes the conversion of DNA methylation to 5-hydroxymethylcytosine (Supplemental Figure 3C). The same pattern was not seen for the *TET2* and *TET3* DNA demethylases in this dataset (Supplemental Figure 3D).

Next, we examined *PROX1* promoter DNA methylation in PDX samples. The two ARPC PDXs that do not express *PROX1* (Supplemental Table 1) harbored elevated levels of *PROX1* promoter DNA methylation (Figure 3C). Conversely, *PROX1*-expressing NEPC PDXs (Supplemental Table 1) had reduced DNA methylation levels in this differentially methylated region (Figure 3C). We also examined *PROX1* promoter DNA methylation in time-course samples taken from the LTL331/331R NEPC transdifferentiation model. We observed a loss in *PROX1* promoter DNA methylation with concomitant increased *PROX1* expression as tumors progressed from ARPC to NEPC (Figure 2, E and F, and Figure 3C). Moreover, *PROX1* upregulation coincided with upregulation of *TET1*, *TET2*, and *TET3* as the LTL331 tumors progressed to NEPC (Supplemental Figure 3E).

To confirm the DNA methylation findings, we used methylation-specific PCR (MSPCR) to amplify the *PROX1* promoter region implicated in our WGBS studies. ARPC (LuCaP 77 and LTL331) and amphicrine (LuCaP 77CR) PDXs harbored a pattern of hemimethylation with predominantly methylated alleles; an AR activity–low (LuCaP 136) PDX harbored a pattern of hemimethylation with predominantly unmethylated alleles, and NEPC (LuCaP 173.1 and LTL331R) and DNPC (LuCaP 173.2A) PDXs that express high *PROX1* (Supplemental Table 1) did not show any methylation signal (Figure 3D). The same pattern was also observed in ARPC versus DNPC and NEPC cell models (Figure 3D). These results with hemimethylation of the *PROX1* promoter but absent expression suggest that DNA methylation may contribute to *PROX1* silencing in ARPC and amphicrine tumors but that additional regulatory mechanisms may also contribute to *PROX1* repression.

To determine the functional role of the DNA methylation changes we observed in *PROX1*-negative models, we treated ARPC cell lines with the DNA methyltransferase inhibitor 5-aza-2′-deoxycytidine (dAza) (29). Treatment of ARPC cells with dAza increased *PROX1* expression (Figure 3E). Gene re-expression coincided with a reduction in the methylated MSPCR amplicon and a concomitant increase in the unmethylated amplicon (Figure 3F). These data strongly suggest that DNA methylation is an important contributor to *PROX1* expression regulation. Finally, we examined levels of acetylation of lysine 27 on histone H3 (H3K27ac), an activating mark, in published LuCaP ChIP-Seq data (30). NEPC and DNPC PDXs had high levels of H3K27ac at the *PROX1* promoter, while ARPC PDXs did not (Supplemental Figure 3F). We also measured levels of H3K27ac in NEPC and ARPC cell lines using ChIP–quantitative PCR. The *PROX1* promoter was marked by high levels of H3K27ac only in NEPC cells but not in ARPC cells (Supplemental Figure 3G). These results further support enhanced *PROX1* transcription through DNA methylation changes as the mechanism for elevated *PROX1* in lineage plasticity tumors.

### PROX1 promotes growth of AR pathway loss and lineage plasticity tumors.

**Figure 4:**
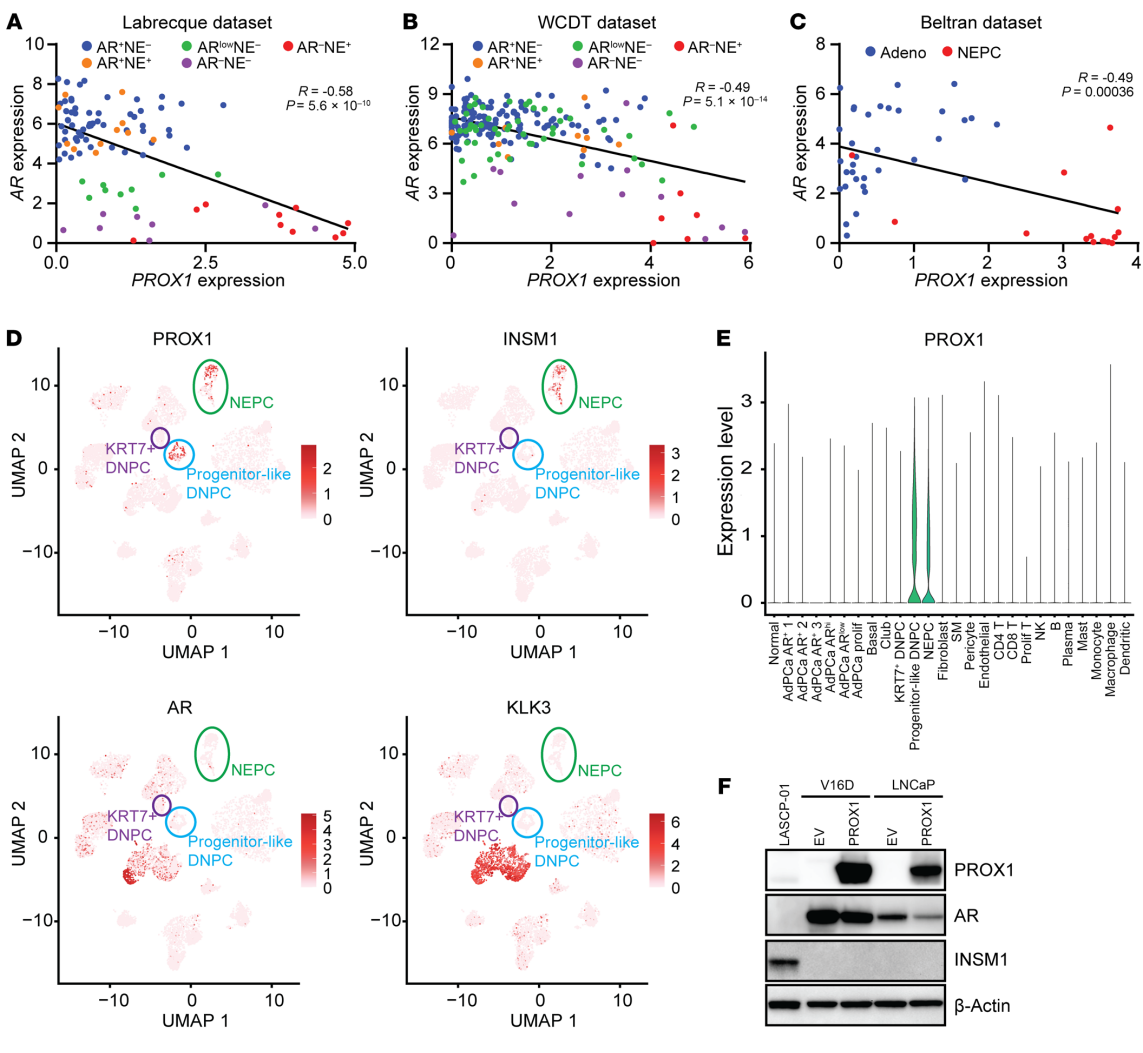
*PROX1* is inversely correlated with the *AR* and is upregulated in progenitor-like DNPC and NEPC tumor clusters. (**A**–**C**) Scatterplots and linear fitted lines of log_2_ TPM expression of *AR* versus *PROX1* in the indicated molecular subtypes of prostate cancer samples from Labrecque et al. 2019 (*n* = 98) (9) (**A**), WCDT (*n* = 210) (8, 22) (**B**), and Beltran et al. 2016 (*n* = 49) (7) datasets (**C**). Pearson’s correlation coefficient (*R*) and *P* values are shown. (**D**) Feature plots of *PROX1*, *INSM1*, *AR*, and *KLK3* expression extracted from scRNA-Seq meta-atlas published in Cheng et al. 2024 (31). Populations of NEPC (green ovals), KRT7^+^ DNPC (purple ovals), and progenitor-like DNPC (blue circles) are marked according to the original publication. (**E**) Violin plot showing the expression level of *PROX1* across different cell populations in the scRNA-Seq meta-atlas of human prostate cancer published in Cheng et al. 2024 (31). *PROX1* is only highly expressed in NEPC and progenitor-like DNPC populations. (**F**) Expression levels of indicated proteins were measured by Western blots in V16D and LNCaP cells transfected with empty vector (EV) or *PROX1* overexpression vector after 72 hours. LASCPC-01 serves as a positive control for PROX1 and INSM1. β-Actin served as loading control.

Having established that *PROX1* is upregulated in tumors that lose AR-dependence and that *PROX1-*expressing lineage plasticity cells often lack *AR* expression (Figures 1 and 2), we further examined that relationship. Examination of bulk RNA-Seq datasets showed a strong inverse correlation between *AR* and *PROX1* expression in CRPC tumors (Figure 4, A–C). Further, we analyzed single-cell RNA-Seq (scRNA-Seq) from a recent report describing 2 previously uncharacterized AR-negative, *KLK3*-negative DNPC populations — one population marked by *KRT7* and another “progenitor-like” DNPC population (31). *PROX1* was highly expressed in the progenitor-like DNPC cell population that was *KRT7* negative but not in the *KRT7*-positive DNPC cell population (Figure 4, D and E, and Supplemental Figure 4A). *PROX1* was also highly expressed in a separate NEPC tumor cell population that was *AR* and *KLK3* negative but positive for NEPC markers, including *INSM1* (Figure 4, D and E). The existence of a *PROX1*-positive DNPC progenitor population further demonstrates that *PROX1* upregulation is not sufficient to induce NEPC lineage plasticity.

*PROX1* expression and *AR* expression are inversely correlated (Figure 4). Furthermore, *PROX1* is upregulated across the AR-independent lineage plasticity continuum, including in AR activity–low and DNPC patient tumors, PDXs, and cell lines that do not express an NEPC program, suggesting that *PROX1* upregulation alone is insufficient to initiate NEPC lineage plasticity (Figures 1, 2, and 4). Therefore, we sought to determine whether PROX1 and the AR negatively regulate each other’s expression. First, we transiently overexpressed *PROX1* in two ARPC cell lines (V16D and C4-2B). *PROX1* overexpression led to reduced *AR* mRNA expression that coincided with reduced AR protein expression (Figure 4F and Supplemental Figure 4B). Next, to determine whether the AR negatively regulates *PROX1*, we examined RNA-Seq data after enzalutamide treatment of three AR-positive models (32). While enzalutamide suppressed expression of the AR target gene *KLK3*, enzalutamide did not increase *PROX1* expression in these *PROX1*-negative cell lines (Supplemental Figure 4C). We also examined RNA-Seq from LNCaP cells harboring stable knockdown of the *AR*, called APIPC cells (3). *AR* knockdown did not increase *PROX1* expression (Supplemental Figure 4D). These data suggest that while PROX1 may negatively regulate *AR* expression, the AR does not appear to negatively regulate *PROX1* expression. Rather, DNA methylation may be a more important mediator of *PROX1* silencing (Figure 3).

We next examined the effects of *PROX1* overexpression on induction of markers of NEPC differentiation. *PROX1* overexpression in V16D and C4-2B cells led to a modest increase in transcript levels of the NEPC marker *INSM1* (Supplemental Figure 4E). However, the levels of INSM1 transcripts induced by *PROX1* overexpression were multiple orders of magnitude lower than the expression levels seen in the NEPC model LASCPC-01 (Supplemental Figure 4E). Importantly, Western blot analysis of cell lysates from the same *PROX1* overexpression experiments failed to detect INSM1 protein expression (Figure 4F), likely because of the low levels of *INSM1* transcripts induced. *PROX1* overexpression led to no change or minimal change in expression of additional NEPC markers — *SYP* or *CHGA* (Supplemental Figure 4E). These results corroborate the expression pattern seen in patient tumors, wherein PROX1 is upregulated in AR activity–low and DNPC tumors that lack NEPC differentiation (Figure 1), and suggest that *PROX1* overexpression alone is insufficient to promote NEPC lineage plasticity.

**Figure 5:**
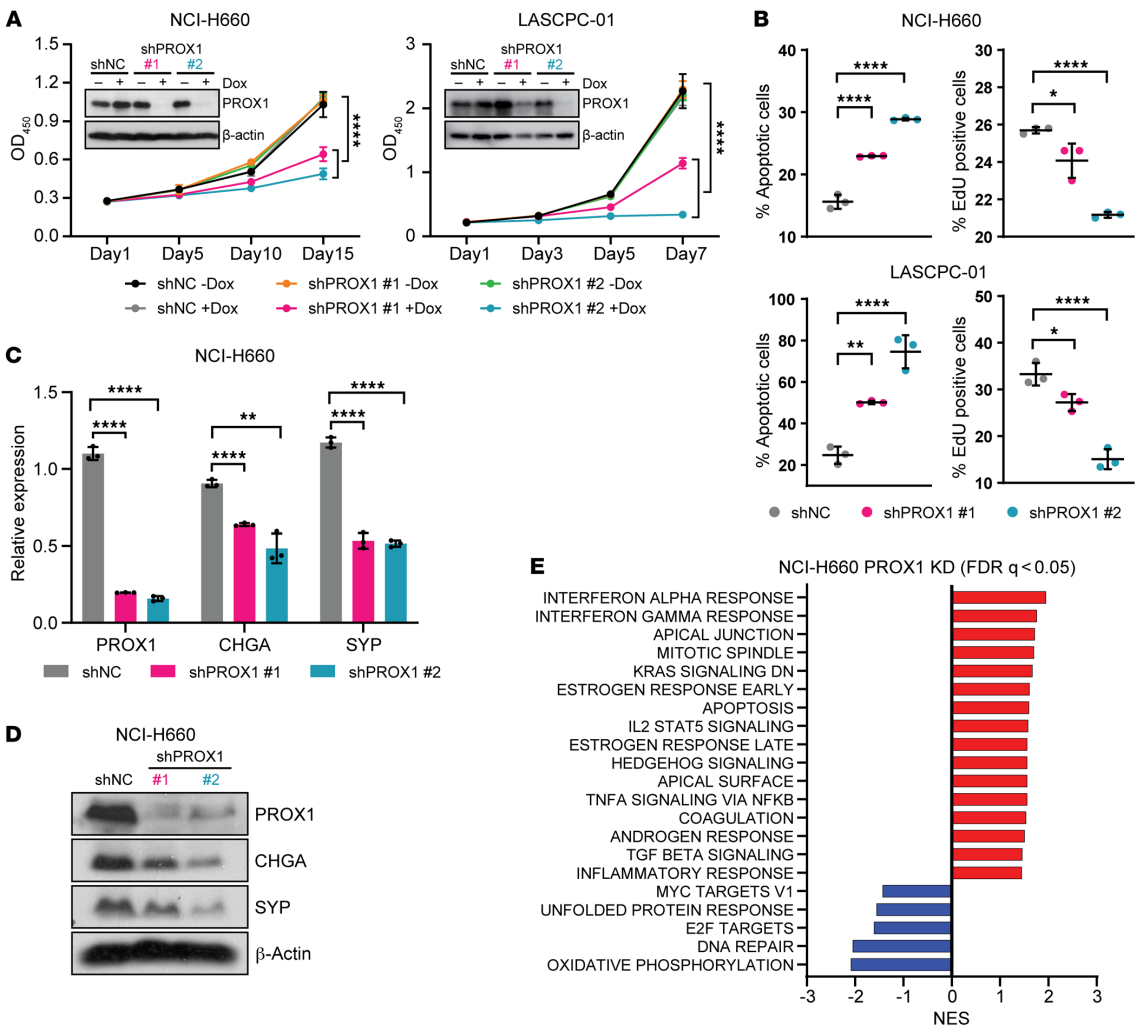
PROX1 promotes differentiation and survival of NEPC models. (**A**) *PROX1* knockdown with 2 different doxycycline-inducible (Dox-inducible) shRNAs versus non-targeted control (NC) shRNA was performed in NCI-H660 and LASCPC-01 cells. Cell viability was measured by CCK-8 assays at the indicated time points. Data are reported as the mean ± SD (*n* = 6 for NCI-H660, *n* = 4 for LASCPC-01). PROX1 knockdown efficiency for each shRNA was confirmed by Western blotting. β-Actin served as loading control. For statistical analysis, shNC +Dox was compared with each shRNA +Dox by Student’s *t* test with Welch’s correction. ****P < 0.0001. (**B**) Apoptosis and EdU assays were performed using cells with Dox (1 μg/mL) treatment for 8 days. Data are reported as the mean ± SD (*n* = 3). For statistical analysis, 1-way ANOVA with Dunnett’s multiple-comparison test was performed. *P < 0.05; **P < 0.01; ****P < 0.0001. (**C**) RT-qPCR was used to measure expression of the indicated NEPC markers (*CHGA* and *SYP*) or *PROX1* in NCI-H660 cells. β-Actin served as housekeeping control. Data are reported as the mean ± SD (*n* = 3). For statistical analysis, unpaired *t* test with Holm-Šidák method for multiple comparison was performed. **P < 0.01; ****P < 0.0001. (**D**) Western blots were used to measure expression of the indicated NEPC markers (CHGA and SYP) or PROX1 in NCI-H660 cells. β-Actin served as loading control. (**E**) Gene set enrichment analysis was performed on RNA-Seq samples (*n* = 2) from Dox-inducible shPROX1 or shNC NCI-H660 cells harvested after 8 days of Dox (1 μg/mL) induction.

Next, we sought to determine whether PROX1 plays a role in maintaining the NEPC phenotype once it is established and promoting NEPC cell survival as has been described in small cell lung cancer (19). Therefore, we suppressed *PROX1* using doxycycline-inducible shRNA. *PROX1* knockdown in NEPC NCI-H660 and LASCPC-01 cells led to reduced cell growth, and the degree of growth suppression correlated with the degree of PROX1 depletion (Figure 5A). *PROX1* knockdown reduced proliferation while increasing apoptosis (Figure 5B). Furthermore, *PROX1* knockdown reduced expression of NEPC markers (Figure 5, C and D, and Supplemental Figure 5, A and B). In summary, our data suggest that PROX1 upregulation alone is insufficient to promote NEPC lineage plasticity, matching the pattern seen in endogenous PROX1-expressing AR activity–low and DNPC patient tumors, PDXs, and cell lines (Figures 1, 2, and 4). However, our results do suggest that PROX1 is important for maintaining the differentiation state and promoting survival of NEPC tumors.

To further understand the pathways modulated by PROX1, we performed RNA-Seq following *PROX1* knockdown in NCI-H660 cells. *PROX1* knockdown modulated several pathways linked to NEPC lineage plasticity (4, 7). Androgen response, estrogen response, inflammatory response, and apoptosis were all activated by *PROX1* knockdown, while Myc signaling and E2F signaling were suppressed (Figure 5E and Supplemental Table 2).

Because PROX1 is also upregulated in DNPC tumors (Figures 1 and 2), we examined its functional role in DNPC as well. *PROX1* suppression with shRNA in the MSKPCa16 DNPC organoid significantly reduced growth and induced apoptosis (Supplemental Figure 5C). These results further demonstrate the importance of PROX1 not only in NEPC but also in DNPC.

### HDAC inhibition blocks PROX1 expression and growth of NEPC and DNPC tumors.

**Figure 6:**
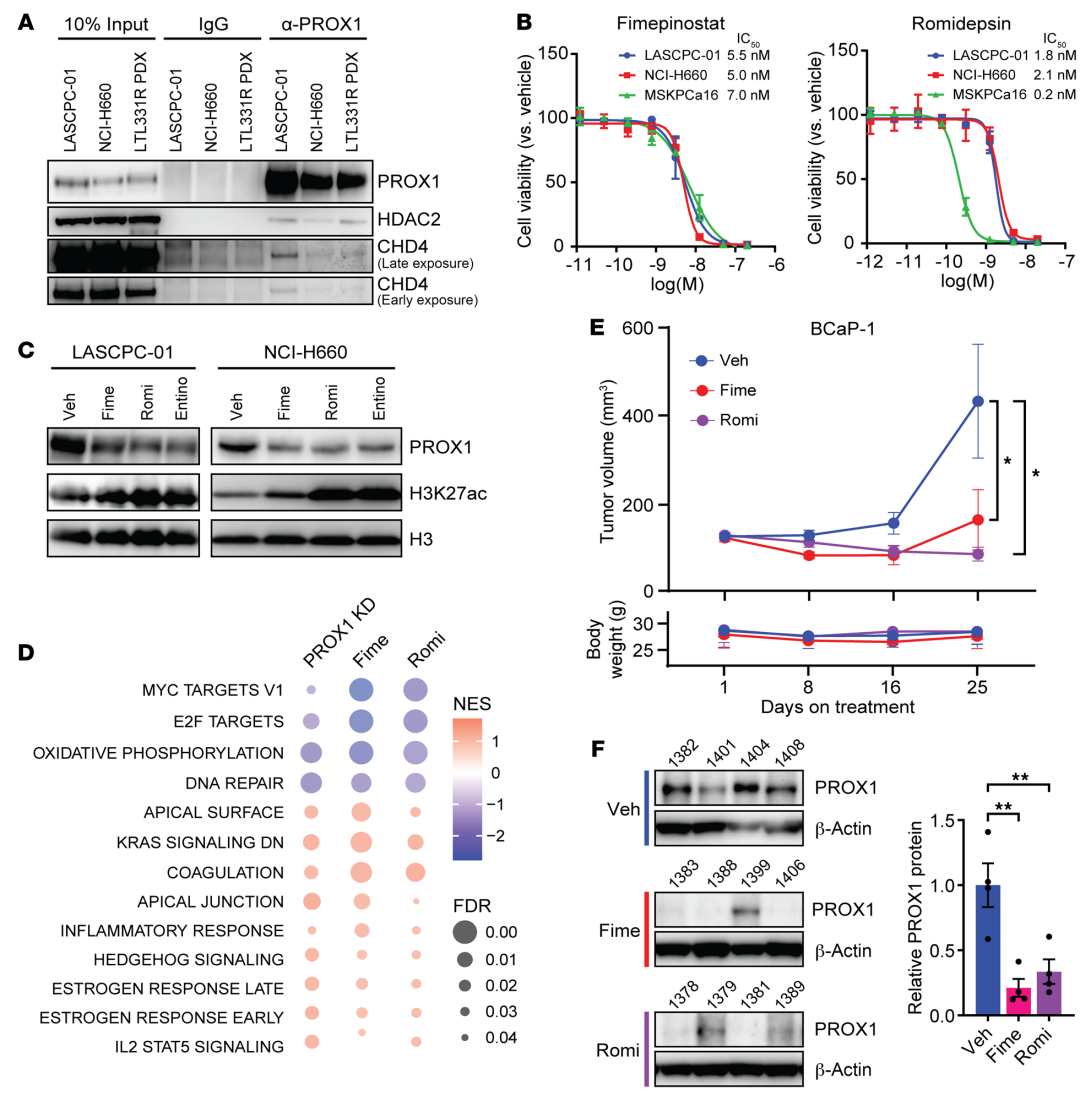
HDAC inhibition blocks PROX1 expression and growth of NEPC and DNPC models. (**A**) A PROX1 antibody was used to pull down PROX1 in LASCPC-01 cells, NCI-H660 cells, and an LTL331R PDX tumor. IgG was used as a negative control. Western blots were used to measure the indicated proteins. Input samples were included as endogenous control. (**B**) The indicated cell lines were treated with fimepinostat (left) and romidepsin (right), and cell viability was measured by CellTiter-Glo assays. IC_50_ values are shown. Data are reported as the mean ± SD (*n* = 4). (**C**) The indicated cell lines were treated with 5 nM fimepinostat, 2 nM romidepsin, or 500 nM entinostat for 48 hours. The indicated proteins were measured by Western blot. Histone H3 serves as loading control. (**D**) Overlapping pathways that change with PROX1 knockdown from Figure 5E or treatment with the HDAC inhibitor fimepinostat (Fime) or romidepsin (Romi) in NCI-H660 cells using RNA-Seq data from Zhang et al. 2023 (34) are presented as a bubble plot. (**E**) NSG mice were implanted with DNPC BCaP-1 PDXs and treated with vehicle, fimepinostat (75 mg/kg orally, 5 times per week), or romidepsin (1.5 mg/kg intraperitoneally, 2 times per week). Tumor volume (top) and mouse body weight (bottom) are shown. Data are reported as the mean ± SEM (*n* = 9 for vehicle and romidepsin, *n* = 6 for fimepinostat). Statistical significance was calculated using 2-way ANOVA with Dunnett’s multiple-comparison test. *P < 0.05. (**F**) Left: Protein was extracted from 4 endpoint PDX tumors from vehicle-, fimepinostat-, or romidepsin-treated mice and probed with the indicated antibodies by Western blot. Right: Quantification of PROX1 protein from the Western blot is presented as a bar plot. Data are reported as the mean ± SEM (*n* = 4). Statistical significance was calculated by 1-way ANOVA with Dunnett’s multiple-comparison test. **P < 0.01.

PROX1 is a transcription factor that is not targetable with standard drug development approaches. To identify important PROX1 coregulators that may be targetable, we immunoprecipitated PROX1 from LASCPC-01 NEPC cells and then performed mass spectrometry. Among the top interacting factors were members of the NuRD repressor complex (33) — including HDAC1, HDAC2, and CHD4 (Supplemental Figure 6A and Supplemental Table 3). We next immunoprecipitated HDAC2 and found considerable overlap between proteins from the PROX1 and HDAC2 pull-downs (Supplemental Figure 6A and Supplemental Table 3). We confirmed these results using IP–Western blots in additional models (Figure 6A). These interaction data suggested that targeting HDACs, for which several HDAC inhibitors (HDACis) exist, may be a promising approach to block PROX1 function in lineage plasticity tumors. Indeed, our prior work showed that HDACi blocks growth of NEPC tumors (34), though the mechanisms accounting for that were not fully characterized.

We confirmed that HDACis were active in PROX1-expressing NEPC NCI-H660 and LASCPC-01 cells and DNPC MSKPCa16 organoids in vitro (Figure 6B and Supplemental Figure 6B). These HDACis include romidepsin, which was approved for cutaneous T cell lymphoma (35); entinostat, which has been tested in breast cancer (36); and fimepinostat (also known as CUDC-907) (37), a dual-acting synthetic small molecule that inhibits HDACs plus PI3K and that has undergone clinical trials in lymphoma patients. Each HDACi blocked cell growth and PROX1 protein expression, coinciding with on-target increased histone acetylation (Figure 6, B and C, and Supplemental Figure 6, B and C).

*PROX1* mRNA did not decline with HDACi in LASCPC-01 cells (Supplemental Figure 6D). We determined that fimepinostat reduced PROX1 protein half-life in the setting of increased histone acetylation (Supplemental Figure 6E), strongly suggesting that inhibition of HDAC function affects PROX1 protein stability. *PROX1* mRNA did decline in NCI-H660 cells (Supplemental Figure 6D). However, the extent of *PROX1* mRNA decline did not appear to fully explain the PROX1 protein declines observed in NCI-H660 (Figure 6C). Together, these results suggest that HDAC inhibition impacts PROX1 levels both post-transcriptionally and transcriptionally.

To determine the functional importance of *PROX1* suppression for the HDACi antitumor activity, we tested two HDACis in isogenic *PROX1*-knockdown and control cells. The IC_50_ values were higher for both HDACis in *PROX1*-deficient cells (Supplemental Figure 6F), demonstrating the importance of PROX1 for the antitumor activity of HDACis. Importantly, there was a significant overlap of pathways modulated by *PROX1* shRNA and treatment of NCI-H660 with romidepsin or fimepinostat (Figure 6D and Supplemental Table 4), further suggesting that PROX1 suppression contributes to the antitumor activity of HDACis.

Our prior work demonstrated that fimepinostat suppressed the growth of LuCaP 145.1 and LuCaP 208.1 NEPC PDXs implanted in mice (34). We measured PROX1 protein expressed in these PDXs and determined that fimepinostat-treated tumors had significantly lower PROX1 protein levels (Supplemental Figure 6G). Finally, we sought to evaluate HDACis in DNPC. Treatment of the DNPC BCaP-1 PDX with fimepinostat and romidepsin showed significant growth suppression (Figure 6E), and HDACi treatment reduced PROX1 levels (Figure 6F). As in our prior NEPC PDX experiments (34), HDACi treatment was well tolerated, and there was no change in mouse body weight (Figure 6E).

## Discussion

The incidence of lineage plasticity appears to be increasing since the more widespread adoption of ARPIs for the treatment of prostate cancer (3, 4, 6, 9). The earliest-acting factors influencing prostate cancer lineage plasticity have not yet been fully characterized. Herein, we focused on *prospero homeobox 1* (*PROX1*), which we found to be strongly linked to the emergence of lineage plasticity early on and which we determined to be critical for promoting important cancer hallmarks in tumors undergoing lineage transitions.

We found that *PROX1* is upregulated early in the lineage plasticity continuum in tumors that have reduced AR activity but that have not already undergone NEPC lineage plasticity. These include AR activity–low tumors and DNPC tumors. Importantly, we determined that ARPI treatment with enzalutamide in patients leads to *PROX1* upregulation in tumors that convert from ARPC to DNPC, suggesting the importance of *PROX1* upregulation in ARPI-induced DNPC lineage plasticity. These results implicating PROX1 in ARPI-induced lineage plasticity were further corroborated by our studies using the LTL331/331R castration-induced NEPC transdifferentiation model. That model allowed us to determine that *PROX1* was upregulated early on after castration before the induction of NEPC markers. Furthermore, by examining scRNA-Seq data, we determined that PROX1 is upregulated in a progenitor-like DNPC population marked by high expression of the transcription factor SOX2 — a Yamanaka reprogramming factor (38) that is implicated in prostate cancer lineage plasticity (39, 40) — but not in a *KRT7*-positive DNPC cell population. These results are in keeping with PROX1’s role in normal neural development as a driver of early neural progenitor cells (13–18) and recent work demonstrating that PROX1 plays a role in regulating lineage plasticity and a fetal progenitor state in colorectal cancer (41), and they suggest that PROX1 induction may facilitate a cell state that is more conducive to or tolerant of differentiation change to terminal states such as progenitor-like DNPC or NEPC.

Importantly, we did not identify cells expressing PROX1 at baseline in the LTL331 model before castration, and our prior work strongly suggests that LTL331R and LTL331 have highly conserved genomic alterations (24). Furthermore, we did not identify cells expressing PROX1 at baseline in a patient whose progression tumor biopsy after AR inhibition was most consistent with DNPC. Exhaustion of tissue samples from additional patients in our prior study (6) profiling ARPC tumors that underwent DNPC conversion after enzalutamide precludes us from determining whether preexisting PROX1-high cells were present at baseline in some of these patients. However, our results suggest that adaptive, epigenetic induction of *PROX1*, rather than selection for a preexisting, genetically distinct *PROX1*-positive subpopulation, may account for the increase in *PROX1*-expressing cells.

Prior work suggests that *PROX1* is upregulated in *RB1*-knockout/*NMyc*-overexpressing NEPC mouse tumors and that poorly differentiated sections of these tumors lose *PROX1* DNA methylation (42). By examining patient tumors, PDXs, and cell lines, we determined that increased DNA methylation at the *PROX1* promoter is strongly linked to *PROX1* mRNA suppression. Active DNA demethylation by the ten-eleven translocation (TET) enzymes coincides with increased 5-hydroxymethylation (28). In examining our prior 5-hydroxymethylation data (27), we found a strong direct correlation between *PROX1* promoter 5-hydroxymethylation and *PROX1* expression. We also found that upregulation of several *TET* demethylases correlated with *PROX1* upregulation. A recent study demonstrates that zinc finger protein 397 (ZNF397) deficiency promotes the transition from a luminal program to a TET2-driven lineage plasticity program (43). Another recent report demonstrates that the tumor suppressor gene *LBK1* (*STK11*) is lost in AR-independent prostate cancer subtypes (44). *LBK1* loss in a murine model promotes activation of several TET DNA demethylases, leading to global DNA hypomethylation and an AR-independent phenotype (44). Thus, genetic changes in *RB1* or *NMyc* or downregulation of *ZNF397* or *LBK1* are possible explanations for how *PROX1* — in addition to other genes critical for lineage plasticity — becomes demethylated and upregulated.

In further support of DNA methylation as one regulatory mechanism for *PROX1* suppression, treatment with the DNA methyltransferase inhibitor dAza led to re-expression of *PROX1* and demethylation of the *PROX1* promoter. Interestingly, several PROX1-negative ARPC cell lines appeared to have hemimethylation of *PROX1*. Indeed, a recent report demonstrates that the polycomb protein EZH2 may repress PROX1 (45), suggesting that repressive histone methylation may be responsible for silencing the unmethylated alleles in *PROX1*-negative tumors.

Our examination of bulk and single-cell RNA-Seq data established a strong inverse relationship between *PROX1* and *AR* expression or AR signaling in CRPC. Indeed, we determined that *PROX1* overexpression in ARPC models led to reduced AR mRNA and protein, suggesting that *PROX1* upregulation may contribute to AR pathway loss. However, as opposed to a recent report, we did not find that AR suppression with enzalutamide or with *AR* knockdown increased *PROX1* expression (21). Combined with our results implicating the role of DNA methylation and 5-hydroxymethylation in *PROX1* regulation, these results suggest that *PROX1* upregulation is not a direct consequence of suppression of AR function.

A prior report linked PROX1 overexpression in ARPC to NEPC lineage plasticity (21). However, in keeping with human tumor data demonstrating that *PROX1* is expressed in AR activity–low or DNPC tumors that do not harbor an NEPC differentiation program, our results demonstrate that transient *PROX1* overexpression in ARPC cells led to a minor increase in expression of the NEPC marker *INSM1* but even less for other NEPC markers (e.g., *SYP* or *CHGA*). Moreover, INSM1 protein expression was not induced by PROX1 overexpression, likely owing to the minimal change in *INSM1* mRNA. We do not know whether longer-term *PROX1* upregulation or choice of different ARPC models might induce more of an NEPC phenotype. It is possible that cooperativity with other transcription factors important for terminal neuronal differentiation (e.g., ASCL1, NEUROD1, NKX2.1) may be necessary (19, 46–49).

Our affinity purification studies demonstrated that HDACs, including HDAC1 and HDAC2, which are targetable, were among the top PROX1-interacting factors. Importantly, members of the HIRA complex (50) were also enriched (including HIRA, UBN1/2, and CABIN1), and we cannot rule out an important cooperative role for PROX1 with those proteins. However, we focused on HDACs because these proteins are targetable with drugs that are in use in the clinic. Indeed, we previously tested HDACis in CRPC patients (51). Importantly, that clinical trial in unselected CRPC patients demonstrated safety and resensitization to AR inhibition (51). However, the impact of HDACis in specific lineage plasticity subtypes was underexplored. In this report, we show that HDACis recapitulated the effects of *PROX1* suppression to reduce cell growth of DNPC and NEPC models, deplete PROX1 protein expression, and modulate expression of key pathways linked to lineage plasticity. These effects were achievable with 3 different HDACis: romidepsin, which blocks HDAC1 and HDAC2 (52); entinostat, which blocks HDAC1, HDAC2, and HDAC3 (53); and fimepinostat, which blocks HDAC1, HDAC2, HDAC3, and HDAC10 (37). While it is unclear which specific HDAC is critical for cooperating with PROX1 or stabilizing its protein expression, it is quite likely that there is redundancy across a number of the HDAC proteins targeted by these inhibitors for the effects we observed.

Our RNA-Seq experiments after *PROX1* knockdown demonstrated modulation of several pathways linked to lineage plasticity (upregulation of androgen and estrogen response; downregulation of MYC, E2F1 targets) (4, 7, 47, 54) — many of which were modulated in the same way by treatment with romidepsin or fimepinostat. Moreover, our isogenic *PROX1-*knockdown versus control cell line experiments demonstrated that *PROX1* expression modulates HDACi sensitivity. These results suggest that PROX1 suppression — at least in part — contributes to the antitumor activity of HDACis. Building on our prior results with HDACis in NEPC (34), our new results suggest that HDACis may be a promising approach to block PROX1 in DNPC tumors, especially given the good animal tolerance and patient tolerance of HDACis in a prior clinical trial (51).

There are several limitations of our report, some of which have been outlined above. PROX1 is upregulated in both DNPC and NEPC, suggesting that PROX1 upregulation may be necessary but not sufficient to promote NEPC lineage plasticity. It is unclear whether specific chromatin states or cooperating factors are necessary to facilitate NEPC lineage plasticity alongside PROX1, an area we are actively investigating. Finally, HDACi depleted PROX1 protein in the models tested — an effect explained in part by reduced PROX1 protein stability. However, reduced *PROX1* mRNA also appeared to contribute to PROX1 protein reduction in some models (e.g., NCI-H660). We are currently exploring these mechanisms, but the fact that these effects were recapitulated by multiple HDACis strongly suggests that PROX1 suppression is an on-target effect of HDAC inhibition.

In summary, our work suggests that PROX1 is a factor activated early in lineage plasticity and a target of interest across the prostate cancer lineage plasticity continuum worthy of further study. Identifying PROX1 upregulation in tumors may help to stratify patients at greatest risk of eventually developing more terminally differentiated forms of prostate cancer such as DNPC or NEPC. Furthermore, our results suggest that PROX1 targeting through HDACis is a rational approach to treat tumors across the prostate cancer lineage plasticity continuum that have upregulated PROX1 and lost AR reliance.

## Methods

Details of experimental procedures for immunoprecipitation and mass spectrometry are included in Supplemental Methods.

### Sex as a biological variable.

Our study exclusively examined male patients and mice because the disease modeled is only relevant in males.

### Cell lines and PDXs.

V16D (shared by Amina Zoubeidi, University of British Columbia, Vancouver, British Columbia, Canada), LASCPC-01 (shared by Owen Witte, UCLA, Los Angeles, California, USA), C4-2B, and LNCaP were cultured as described previously (32, 54, 55). MSKPCa16 organoids (shared by Yu Chen, Memorial Sloan Kettering Cancer Center, New York, New York, USA) were cultured as described previously (56). NCI-H660 cells (CRL-5813) were purchased from ATCC and cultured according to ATCC’s recommendation. LuCaP PDXs were maintained as described previously (57). All cell lines were validated with STR DNA fingerprinting by Genetica Cell Line Testing (a LabCorp brand) and regularly tested for mycoplasma contamination by the MycoAlert Mycoplasma Detection Kit (Lonza catalog LT07-318).

### Chemicals.

Decitabine (5-aza-2′-deoxycytidine [dAza]) (catalog HY-A0004), fimepinostat (catalog HY-13522), and romidepsin (catalog HY-15149) were purchased from MedChemExpress, and entinostat was purchased from Selleck Chemicals (catalog S1053). All drugs were dissolved in DMSO at indicated concentration. DMSO was used as vehicle control. Doxycycline hyclate (MilliporeSigma catalog D9891) dissolved in water was used for experiments with doxycycline-inducible constructs at a final concentration of 1,000 ng/mL.

### Survival analyses with PROX1 expression.

Tumor samples from patients with metastatic CRPC were profiled using capture RNA sequencing as previously described from 2 cohorts, WCDT (8, 22) and Abida et al. (23). *PROX1* (ENSG00000117707) expression measured in transcripts per million mapped reads (TPM) for the WCDT cohort or measured in reads per kilobase per million mapped reads (RPKM) for the Abida et al. cohort was ranked across patients. Patients were split into groups by *PROX1* expression quartiles. Overall survival from the time of tumor biopsy acquisition was compared between groups for both cohorts. Survival analyses were performed with survival and survminer packages in R, and survival probability was visualized with Kaplan-Meier curves. Hazard ratios were modeled using the Cox proportional hazards model, and differences were tested using log-rank tests.

### Immunohistochemistry.

For all IHC PROX1 protein detection experiments, 5 μm tissue sections were stained using a previously validated PROX1 antibody (clone D2J6J, rabbit mAb 14963, Cell Signaling Technologies). Briefly, formalin-fixed, paraffin-embedded (FFPE) sections were deparaffinized and rehydrated according to standard protocols. Antigen retrieval was performed by steaming for 45 minutes in Target Retrieval Solution (S1700, Agilent). The slides were then washed and equilibrated in TBS-Tween buffer (Sigma-Aldrich) for 10 minutes. PROX1 antibodies were applied at a dilution of 1:50, and immunocomplexes were visualized using the UltraVision Quanto detection system (TL060QHD, Fisher Scientific) followed by the Invitrogen Biotin XX Tyramide SuperBoost kit (B40931, Fisher Scientific). All tissue sections were counterstained with hematoxylin, and the slides were digitized using a Ventana DP 200 Slide Scanner (Roche). The slides were reviewed by 2 pathologists, and immunoreactivities were scored in a blinded manner using a previously established H-score system, where the optical density level (0 for no brown color, 1 for faint and fine brown chromogen deposition, and 2 for prominent chromogen deposition) was multiplied by the percentage of cells at each staining level, resulting in a total H-score (range 0–200) for each core.

### Multiplex immunofluorescence.

Sequential multiplex immunofluorescence study of LuCaP 136 was performed on archival FFPE tissue as previously described (6). The following antibodies were used in sequence at the specified dilutions: PROX1 (1:50; Cell Signaling Technology catalog 14963), PSA (1:50; Cell Signaling Technology, 2475T), INSM1 (1:50; Santa Cruz Biotechnology, sc-271408), and AR (1:100; Cell Signaling Technology, 5153T). Signal amplification was achieved by incubation of the slides with PowerVision Poly-HRP Anti-Rabbit or Anti-Mouse secondary antibodies (Leica), followed by the application of different fluorophore-tyramide conjugates (Thermo Fisher Scientific). After each staining step, antibody stripping was performed by steaming for 20 minutes in either Citrate Buffer (H-3300-250, Vector Laboratories) or Target Retrieval Solution (S1700, Agilent). The slides were then mounted with ProLong (Thermo Fisher Scientific), imaged using a Nikon Eclipse E800 microscope, and analyzed with QuPath (v0.3.0) (58).

### Chromatin immunoprecipitation.

ChIP experiments were performed as described previously (59, 60) using anti-H3K27ac (Active Motif catalog 39133) or rabbit IgG (Millipore catalog 12-370). Briefly, 10 million formaldehyde–cross-linked cells were lysed and sheared with the Bioruptor Pico (Diagenode). Four micrograms of antibody was used to immunoprecipitate chromatin from 2 million cells per ChIP overnight at 4°C. The DNA-protein-antibody complexes were pulled down with 30 μL protein A/G magnetic beads (Thermo Fisher Scientific catalog 26162). After washing, DNA was extracted by 10% Chelex-100 resin (Bio-Rad catalog 1421253) and digested with Proteinase K (Invitrogen catalog AM2546). Quantitative PCR was performed with SYBRGreen PCR master mix (Thermo Fisher Scientific catalog 4312704) using a Quantstudio 3 or Quantstudio 5 thermocycler (Applied Biosystems). Primer sequences are provided in Supplemental Table 5.

### Cell viability assay.

Cell viability at different time points was evaluated using the Cell Counting Kit-8 (CCK-8) assay (Dojindo Laboratories catalog CK04) according to the protocol of the manufacturer. In brief, cells were seeded with doxycycline in 96-well plates and incubated overnight. On the designated day for measuring viability, 20 μL of CCK-8 reagent was added to each well containing 200 μL of medium (1:100 ratio). The plates were then incubated for 3 hours at 37°C in a 5% CO_2_ atmosphere to facilitate the reaction. After incubation, absorbance was measured at 450 nm to quantify cell viability, providing an indication of metabolic activity and, consequently, the overall growth of viable cells.

### Dose-response experiments.

For dose-response experiments, the indicated cells were treated in biological triplicate for 72 hours with a 7-point, 5-fold dilution series from 10 mM of the indicated drugs in DMSO. Cell viability was assessed using the CellTiter-Glo 3D Cell Viability assay (Promega catalog G9683). Dose-response was normalized to the vehicle-treated growth rate and fitted with a logistic curve as previously described (61).

### Apoptosis assay.

Apoptosis was measured using annexin V (BD Biosciences catalog 550475) according to the manufacturer’s instructions. Briefly, cells at indicated time points were trypsinized, harvested, and stained with 5 μL allophycocyanin-conjugated (APC-conjugated) annexin V and 5 μL propidium iodide (PI) (Sigma-Aldrich catalog P4864) for 15 minutes. The cells were analyzed by LSRFortessa II (BD Biosciences) for APC (annexin V) or PI signal. Unstained and single-stained (annexin V–APC or PI) cells were used to gate stained populations.

### EdU assay.

Cell proliferation was measured by EdU incorporation using the Click-iT Plus EdU Alexa Fluor 647 Flow Cytometry Assay Kit (Thermo Fisher Scientific catalog C10634) according to the manufacturer’s instructions. Briefly, cells at indicated time points were pulsed with 10 μM EdU for 3 hours. Cells were then fixed and permeabilized, and S-phase cells that incorporated EdU were stained with Click-iT Plus detection cocktail. The cells were analyzed by LSRFortessa II (BD Biosciences) for percentage EdU-positive cells. Cells without EdU incorporation were used to gate the EdU-positive population.

### Plasmid transfection.

For overexpression experiments, plasmids were transfected using Lipofectamine 3000 reagent (Thermo Fisher catalog L3000008) per the manufacturer’s recommendations. Cells were harvested 72 hours after transfection and processed for downstream analyses. The *PROX1* expression construct was developed by cloning of *PROX1* cDNA into NotI and XbaI sites of p3XFLAG-CMV-10 vector. Empty vector p3XFLAG-CMV-10 was used as control for overexpression studies. The correct sequence of the *PROX1* expression vector was confirmed by Sanger sequencing.

### Short hairpin sequences used for PROX1 knockdown.

To achieve the knockdown of the *PROX1* gene in MSKPCa16, NCI-H660, and LASCPC-01 cell lines, two shRNA constructs were specifically designed to target the following sequences: shRNA#1, target sequence TTTCCAGGAGCAACCATAATT; and shRNA#2, target sequence AGTACATCAGGAGGATATATG. These shRNA sequences were then cloned into a doxycycline-inducible plasmid, tet-PLKO-puro (21915, Addgene), resulting in the generation of the tet-PLKO-PROX1-shRNA#1 and tet-PLKO-PROX1-shRNA#2 constructs. Stably transfected cells were subsequently treated with 1 μg/mL doxycycline to facilitate gene expression validation and functional assays.

### RNA preparation and quantitative reverse transcription PCR.

After the indicated treatments, RNA was extracted from cells or organoids using the RNeasy Plus Mini Kit (QIAGEN catalog 74034) according to the manufacturer’s protocol. After RNA extraction, 1 μg RNA was reverse-transcribed into cDNA using the High-Capacity cDNA Reverse Transcription kit (Life Technologies catalog 4368814) with random hexamer primers. Quantitative reverse transcriptase PCR (RT-qPCR) was performed using a Quantstudio 5 thermocycler (Applied Biosystems) with the following program: 50°C for 2 minutes, 95°C for 10 minutes, and 40 cycles of 95°C for 15 seconds dissociation, 60°C for 1 minute annealing/extension/read. Ten-microliter single-plex RT-qPCR reactions contained 1× TaqMan Universal PCR Master Mix (Thermo Fisher Scientific catalog 4304437), 1× Primer and TaqMan hydrolysis probe specific to the target tested (Supplemental Table 6), and 10 ng RNA-equivalent cDNA templates. *GAPDH* was used as endogenous control. Data were analyzed with Design and Analysis Software version 1.5.2 (Life Technologies).

### Western blotting.

Western blotting experiments were performed by running protein lysates on SDS-PAGE gels (Thermo Fisher Scientific catalog NP0335BOX) and transferring them onto PVDF membranes as described previously (60). Blots were probed with indicated antibodies and imaged using a Chemidoc MP imaging system (Bio-Rad). Anti-PROX1 (Cell Signaling Technology catalog 14963), anti-H3K27ac (Active Motif catalog 39133), anti-H3 (Millipore catalog 06-755), anti-AR (Millipore catalog 06-680), anti-NCAM1/CD56 (Cell Signaling Technology catalog 3576), anti-CHGA (Cell Signaling Technology catalog 60893), anti-SYP (Santa Cruz Biotechnology catalog sc-17750), anti-INSM1 (Santa Cruz Biotechnology catalog sc-271408), anti-CHD4 (Proteintech catalog 14173-1-AP), anti-GAPDH (Cell Signaling Technology catalog 2118), and anti-actin (Sigma-Aldrich catalog A5441) were used for protein detection by Western blotting.

### Protein half-life assay.

For protein half-life measurement assays, the indicated cell lines treated with vehicle or drug were treated with 200 μg/mL cycloheximide (Sigma-Aldrich catalog C1988) and harvested at indicated time points. Cells were lysed and analyzed by Western blotting as indicated above. The bands from Western blot images were quantified using ImageJ (62).

### Bisulfite conversion and methylation-specific PCR.

Genomic DNA from untreated cells or cells with indicated treatment (DMSO or dAza) was extracted using a DNeasy Blood & Tissue Kit (QIAGEN catalog 69504) and then underwent bisulfite conversion using the EZ DNA Methylation-Gold Kit (Zymo catalog D5006) according to the manufacturer’s protocol. The methylation status of *PROX1* was evaluated using 2 primer sets, methylated (M) and unmethylated (U) (Supplemental Table 7). Methylation-specific PCR (MSPCR) amplification was performed using Platinum II Taq Hot-Start DNA Polymerase Kit (Invitrogen catalog 14966005) according to the manufacturer’s protocol. The PCR conditions were as follows: 94°C for 2 minutes, 35 cycles of (94°C for 15 seconds, 62°C for 15 seconds, and 68°C for 20 seconds), and 68°C for 30 seconds. The PCR products were subjected to electrophoresis on a 3% agarose (Invitrogen catalog 16500-100) gel. Densitometry analysis was performed using NIH ImageJ (62).

### RNA-Seq and pathway analysis.

Total RNA from NCI-H660 non-targeted control shRNA (shNC) or sh*PROX1* treated with 1 μg/mL doxycycline for 8 days was extracted using the RNeasy Plus Mini Kit (QIAGEN) as described above. RNA libraries were prepared and sequenced on the Illumina NovaSeq 6000 platform (Novogene) with a depth of 20 million paired-end 150 bp reads per sample. The sequencing reads were aligned to the GRCh38 reference genome using the HISAT2 aligner (v2.0.5) (https://daehwankimlab.github.io/hisat2/). Gene expression counts were quantified using featureCounts (v1.5.0-p3) (https://subread.sourceforge.net/featureCounts.html). and normalized to fragments per kilobase of transcript per million mapped reads (FPKM) to account for differences in sequencing depth and gene length. Differential expression analysis was conducted using the DESeq2 package (v1.20.0) in R. Genes with an adjusted *P* value ≤ 0.05, as identified by DESeq2, were classified as differentially expressed. Gene set enrichment analysis (GSEA; v4.3.2) was performed using the weighted enrichment statistic on normalized gene counts, with genes ranked by log_2_ fold change.

### In vivo antitumor study.

*The* BCaP-1 PDX model (shared by W. Nathaniel Brennen, John Hopkins University, Baltimore, Maryland, USA) was maintained by serial passaging in 6- to 8-week-old castrated male NSG (NOD.Cg-Prkdc^Scid^Il2rg^tm1Wji^/SzJ) mice (The Jackson Laboratory catalog 005557) as described previously (63). When tumors reached approximately 120 mm^3^, mice were randomized into vehicle or treatment groups and dosed for 3-and-a-half weeks. For the romidepsin arm, mice received 1.5 mg/kg romidepsin in vehicle (PBS), intraperitoneally, 2 times weekly for 3-and-a-half weeks. For the fimepinostat arm, mice received 75 mg/kg fimepinostat in vehicle (30% wt/vol Captisol, MedChemExpress catalog HY-17031), orally, 5 times weekly for 3-and-a-half weeks. Vehicle arm received vehicle of both romidepsin (PBS intraperitoneally, 2 times weekly) and fimepinostat (30% wt/vol Captisol orally, 5 times weekly) arms for 3-and-a-half weeks. Animals were sacrificed and tumors were harvested after 3-and-a-half weeks of treatment. Animals were sacrificed if the tumors reached the humane endpoint according to the approved protocol before treatment completion. Body weight and tumor measurements were recorded weekly.

### Statistics.

GraphPad Prism version 10.2.3 was used for statistical analysis and plotting of graphs. The data from biological replicates are presented as mean ± SD/SEM with number of replicates indicated in respective figure legends. Statistical tests were performed by a 2-tailed Student’s t test, 1-way ANOVA, or 2-way ANOVA, and *P* value less than 0.05 was considered significant. Details for the statistical test performed for individual figures are listed in respective figure legends.

### Study approval.

All the animal studies were performed under the animal protocol (PRO00011266) reviewed and approved by the Institutional Animal Care and Use Committee at the University of Michigan. Metastatic CRPC specimens were collected under the aegis of the Michigan Oncology Sequencing Project (MI-ONCOSEQ) at the University of Michigan (IRB protocol HUM00046018 and HUM00067928) after informed consent was obtained.

### Data availability.

RNA-Seq data and corresponding clinical annotations of tumor samples are available through the Beltran et al. dataset (7), the dataset from the West Coast Dream Team (WCDT) (8, 22), the Labrecque et al. dataset (9), and prostate cancer PDXs from the Coleman et al. dataset (64). DNA methylation and 5-hydroxymethylation data from patients are available from the Zhao et al. dataset (26) and the Sjöström et al. dataset (27), respectively. The methylation datasets from PDXs reported in this article were deposited in the NCBI’s Gene Expression Omnibus (GEO) — LuCaP PDXs (GSE227086) and LTL331 time-course samples (GSE278588). The RNA-Seq dataset for *PROX1*-knockdown data reported in this article was deposited in GEO (GSE277727). Raw data used to prepare graphs in the figures are provided in the Supporting Data Values file. Raw blot images used to prepare Western blot and agarose gel images in the figures are provided in the unedited blot and gel images file.

## Author contributions

JJA, Yuzhuo Wang, ZD, M Shi, and AK designed the research. ZD, M Shi, DL, AK, DK, Yong Wang, CZ, DF, and ER performed experiments. ZD, M Shi, DL, AK, DK, CZ, DF, ER, OAS, WKS, HX, and ZRM acquired data. RAP, ES, XD, AMU, and MCH performed IHC and multiplex immunofluorescence and evaluation. BPH, RW, DAQ, M Sjöström, YMH, FZ, ZX, SC, XY, FYF, LZ, RA, VR, AR, KB, RJR, and MPC performed computational analyses. ZD, M Shi, AK, DF, JAY, and JJA analyzed the data. JKL and PSN provided cell line models. CM and EC provided PDX samples. RA, EJS, AMC, and JJA provided patient tumor samples. ZD, M Shi, AK, HNB, JAY, and JJA wrote the manuscript. ZD, M Shi, AK, DF, WKS, ZRM, RW, DAQ, ZX, CM, IC, PSN, EC, MPC, JAY, MCH, Yuzhuo Wang, and JJA revised the manuscript. The order of the co–first authors was determined based on their relative contributions to this study. All authors read and approved the final manuscript.

## Supplementary Material

Supplemental data

Unedited blot and gel images

Supplemental tables 1-7

Supporting data values

## Version 1 06/02/2025

Electronic publication
