# Supplementary material for: PROX1 is an early driver of lineage plasticity in prostate cancer
Source: J Clin Invest. 2025 Jun 2;135(11):e187490. doi: 10.1172/JCI187490 (PMC12126232; doi:10.1172/JCI187490)
Supplement: Supplemental data [file jci-135-187490-s270.pdf]

Supplemental Material

PROX1 is an Early Driver of Lineage Plasticity in Prostate Cancer

Zhi Duan[1,2], Mingchen Shi[3,4,5], Anbarasu Kumaraswamy[1,2], Dong Lin[3,4,5], Dhruv Khokhani[1,2], Yong Wang[3,4,5], Chao Zhang[1,2], Diana Flores[1,2], Eva Rodansky[1,2], Olivia A. Swaim[1,2], William K. Storck[1,2], Hannah Beck[1,2], Radhika A. Patel[6], Erolcan Sayar[6], Brian P. Hanratty[6], Hui Xue[3,4,5], Xin Dong[3,4,5], Zoe R. Maylin[3,4,5], Rensheng Wan[7,8], David A. Quigley[7,9,10], Martin Sjöström[7,8,11,12], Ya-Mei Hu[13,14], Faming Zhao[13,14], Zheng Xia[13,14], Siyuan Cheng[15], Xiuping Yu[15], Felix Y. Feng[7,8,10,*], Li Zhang[7,9], Rahul Aggarwal[7,16], Eric J. Small[7,16], Visweswaran Ravikumar[17], Arvind Rao[2,17,18,19], Karan Bedi[2,20], John K. Lee[21], Colm Morrissey[22], Ilsa Coleman[6], Peter S. Nelson[6,23], Eva Corey[22], Aaron M. Udager[2,24,25], Ryan J. Rebernick[17,24,25], Marcin P. Cieslik[17,24,25], Arul M. Chinnaiyan[2,24,25,26], Joel A. Yates[1,2], Michael C. Haffner[6,23,27], Yuzhuo Wang[3,4,5], Joshi J. Alumkal[1,2,25]

[1]Department of Internal Medicine, University of Michigan, Ann Arbor, Michigan, USA
[2]Rogel Cancer Center, University of Michigan, Ann Arbor, Michigan, USA
[3]Vancouver Prostate Centre, Vancouver, British Columbia, Canada
[4]Department of Urologic Sciences, Faculty of Medicine, University of British Columbia, Vancouver, British Columbia, Canada
[5]BC Cancer Research Institute, BC Cancer, Vancouver, British Columbia, Canada
[6]Division of Human Biology, Fred Hutchinson Cancer Research Center, Seattle, Washington, USA
[7]Helen Diller Family Comprehensive Cancer Center, University of California at San Francisco, San Francisco, California, USA
[8]Departments of Radiation Oncology, University of California at San Francisco, San Francisco, California, USA
[9]Department of Epidemiology & Biostatistics, University of California at San Francisco, San Francisco, California, USA
[10]Department of Urology, University of California at San Francisco, San Francisco, California, USA
[11]Division of Oncology, Department of Clinical Sciences Lund, Faculty of Medicine, Lund University, Lund, Skåne, Sweden
[12]Department of Haematology, Oncology and Radiation Physics, Skåne University Hospital, Lund, Skåne, Sweden
[13]Knight Cancer Institute, Oregon Health & Science University, Portland, Oregon, USA
[14]Department of Biomedical Engineering, Oregon Health & Science University, Portland, Oregon, USA
[15]Department of Biochemistry and Molecular Biology, LSU Health Shreveport, Shreveport, Louisiana, USA
[16]Division of Hematology and Oncology, Department of Medicine, University of California San Francisco, San Francisco, California, USA
[17]Department of Computational Medicine & Bioinformatics, University of Michigan, Ann Arbor, Michigan, USA
[18]Department of Biomedical Engineering, University of Michigan, Ann Arbor, Michigan, USA
[19]Department of Radiation Oncology, University of Michigan, Ann Arbor, Michigan, USA
[20]Department of Biostatistics, School of Public Health, University of Michigan, Ann Arbor, Michigan, USA
[21]Department of Medicine and the Institute for Urologic Oncology, David Geffen School of Medicine, University of California at Los Angeles, Los Angeles, California, USA
[22]Department of Urology, University of Washington, Seattle, Washington, USA
[23]Division of Clinical Research, Fred Hutchinson Cancer Research Center, Seattle, Washington, USA
[24]Department of Pathology, University of Michigan, Ann Arbor, Michigan, USA
[25]Michigan Center for Translational Pathology, Rogel Cancer Center, University of Michigan, Ann Arbor, Michigan, USA
[26]Howard Hughes Medical Institute, University of Michigan, Ann Arbor, Michigan, USA
[27]Department of Laboratory Medicine and Pathology, University of Washington, Seattle, Washington, USA
*Deceased

Authorship note: Zhi Duan and Mingchen Shi are co–first authors. Yuzhuo Wang and Joshi J. Alumkal jointly supervised the work.

Address correspondence to: Dr. Joshi J. Alumkal, Department of Internal Medicine, Hematology/Oncology, University of Michigan, 7312 Rogel Cancer Center, 1500 East Medical Center Dr, Ann Arbor, MI 48109, USA. Telephone: +1.734.936.9868; Email: jalumkal@med.umich.edu.

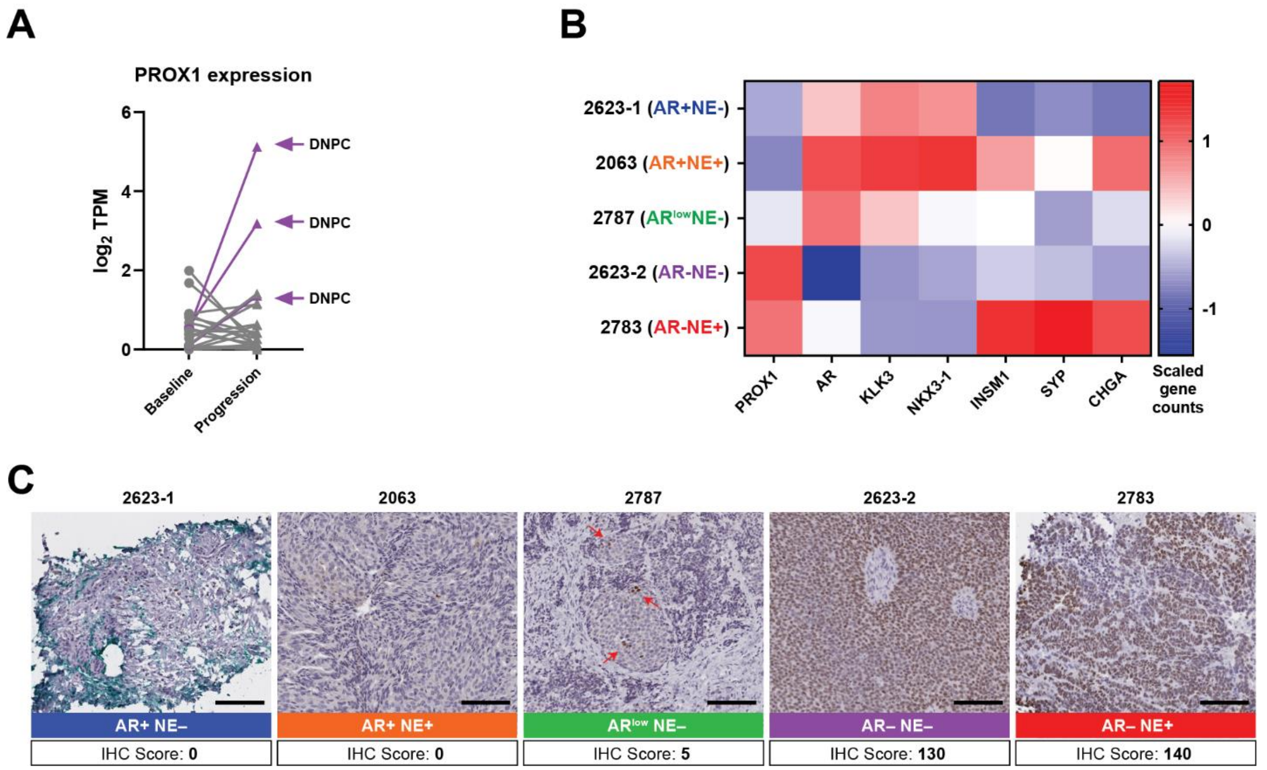

Supplemental Figure 1. *PROX1* is significantly upregulated in biopsy samples from patients that exhibit AR pathway loss and lineage plasticity. (A) *PROX1* mRNA was quantified by RNA-seq in samples from the Westbrook et al. 2022 cohort (Ref. 6). *PROX1* expression was upregulated in all three patient tumors that converted to a DNPC state (marked in purple). **(B)** Expression of indicated genes were quantified by RNA-seq in patient biopsy samples. Molecular subtype of each sample is color coded. The scaled gene counts are indicated in the heatmap. **(C)** Prostate cancer patient tumor biopsy samples corresponding to those shown in panel B were stained with an anti-PROX1 antibody by immunohistochemistry (IHC). Representative fields from these tumor samples and PROX1 IHC scores are shown. Scale bar indicates 100 μm.

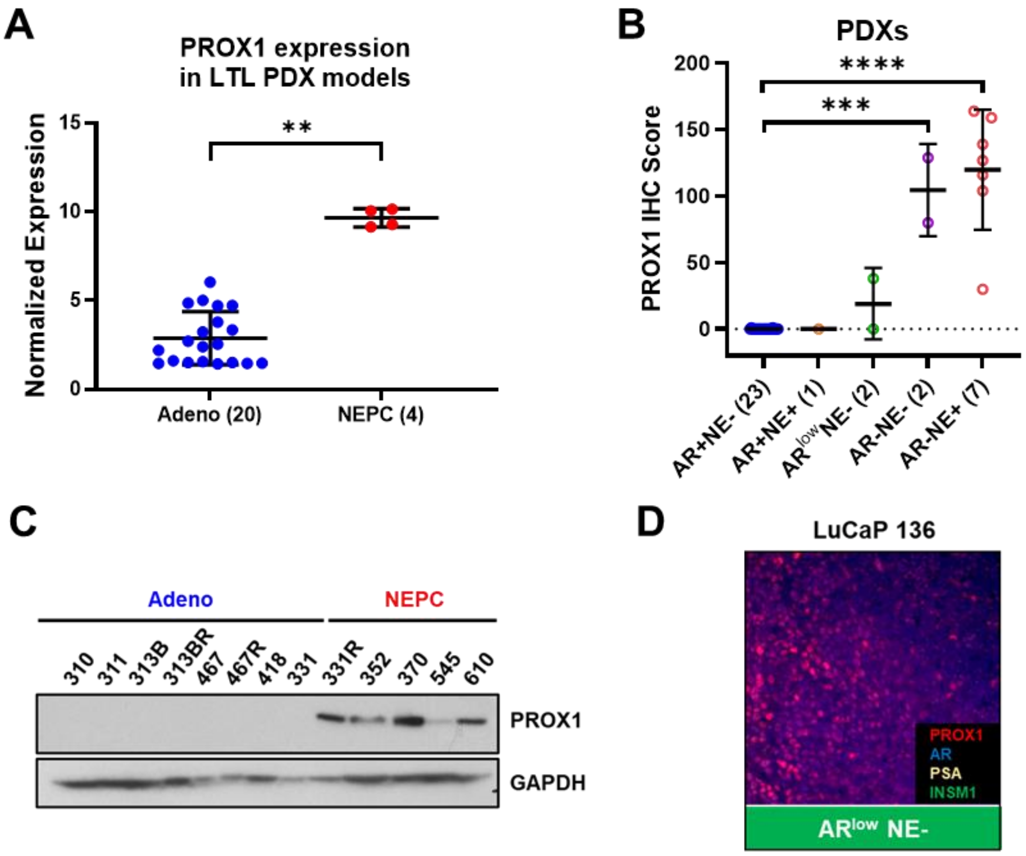

Supplemental Figure 2. *PROX1* is upregulated in patient-derived xenografts that have undergone lineage plasticity. (A) *PROX1* expression was measured by RNA-seq in LTL adenocarcinoma (Adeno, N=20) or NEPC (N=4) PDXs. Data are reported as the mean ± SD. Statistical analysis was performed with unpaired two-sample Wilcoxon test. ***P <0.01.* **(B)** PROX1 IHC scores from prostate cancer patient-derived xenografts (PDXs) representing different molecular subtypes and sample sizes are shown. Data are reported as the mean ± SD. Statistical significance was calculated by unpaired two-sample Wilcoxon test with Benjamini-Hochberg correction for multiple-comparison. ****P <0.001; ****P <0.0001.* **(C)** PROX1 expression in LTL PDXs were measured by Western blots. GAPDH was used as a loading control. **(D)** Multiplex immunofluorescence staining in LuCaP 136 shows expression of indicated proteins: PROX1 (red), AR (blue), PSA (yellow) and INSM1 (green).

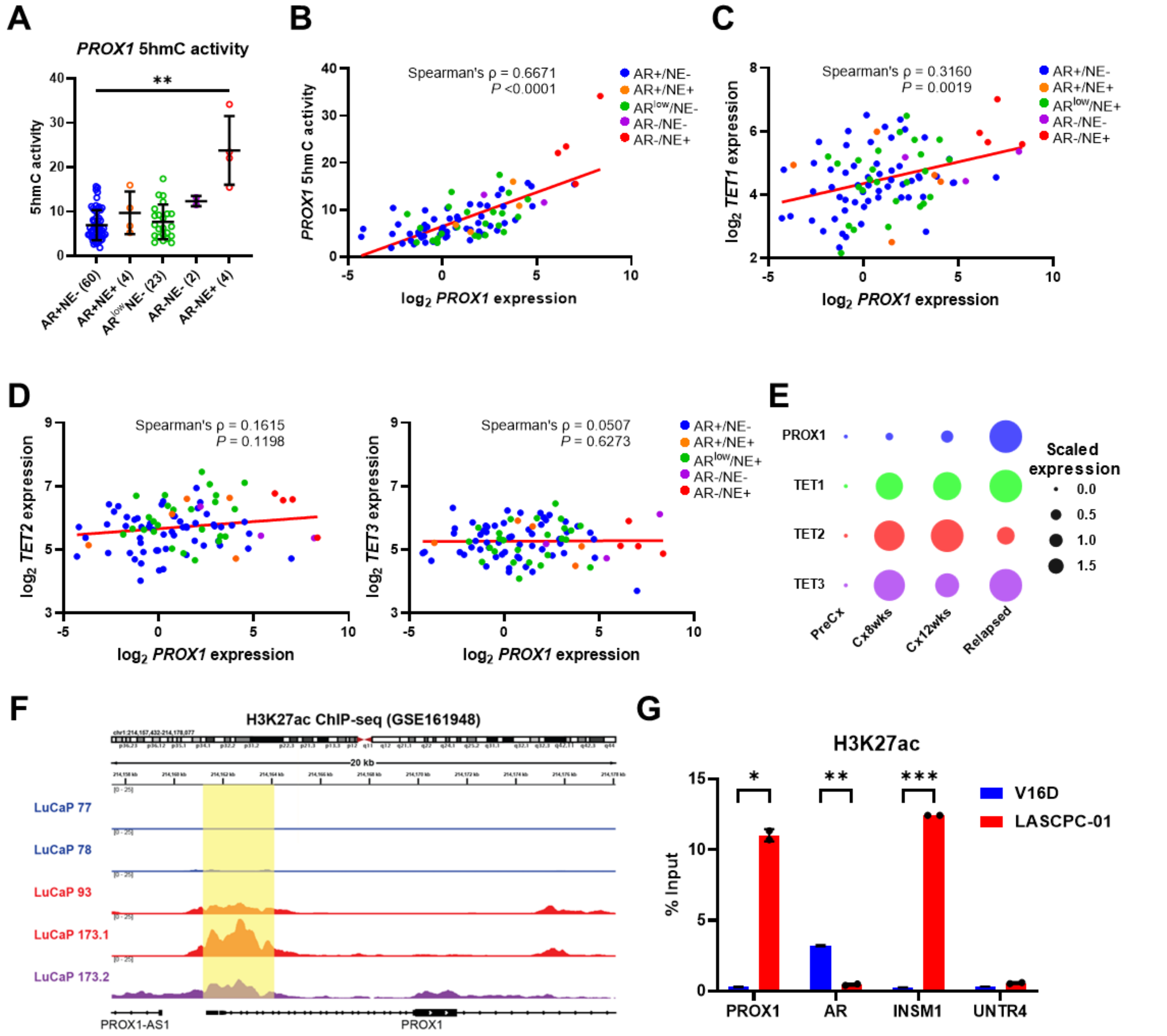

Supplemental Figure 3. *PROX1* expression is epigenetically regulated. (A) *PROX1* 5-hydroxymethylation (5hmC) in prostate cancer patient tumors (N=93) were extracted from Sjöström et al. 2022 dataset (Ref. 27). 5hmC score is shown with the five molecular subtypes and their sample sizes. The DNA 5-hydroxymethylation of *PROX1* is significantly increased in NEPC (AR-NE+) tumors as indicated by *P* value calculated by unpaired two-sample Wilcoxon test with Benjamini-Hochberg correction for multiple-comparison.; ***P*<0.01. **(B)** Scatter plots and linear fitted lines of *PROX1* 5hmC vs. $\log_2$ *PROX1* expression in samples from WCDT dataset (Ref. 22, 27). Spearman's correlation coefficient (ρ) and p values are shown. **(C-D)** Scatter plots and linear fitted lines of $\log_2$ TPM expression of (C) *PROX1* vs. *TET1* (D) *PROX1* vs. *TET2* or *TET3* in the indicated molecular subtypes of prostate cancer samples from WCDT dataset are shown (Ref. 22). **(E)** Bubble plot depicts mRNA expression levels of from RNA-seq in LTL331 PDXs at the indicated time points during progression from LTL331 (PreCx) to LTL331R (Relapsed). **(F)** The genome tracks of H3K27ac at *PROX1* promoter in LuCaP PDXs were extracted from ChIP-seq data from Baca et al. 2021 (Ref. 30). **(G)** H3K27ac at *PROX1* promoter was measured by ChIP-qPCR from V16D and LASCPC-01 cell lines. Normal rabbit IgG control (less than 0.02% Input) was subtracted. UNTR4 in gene desert on human chromosome 4 is used as negative control. Data are reported as the mean ± SD (N=2). For statistical analysis, Student's t test with Welch's correction was performed. *P<0.05; **P<0.01; ***P<0.001.

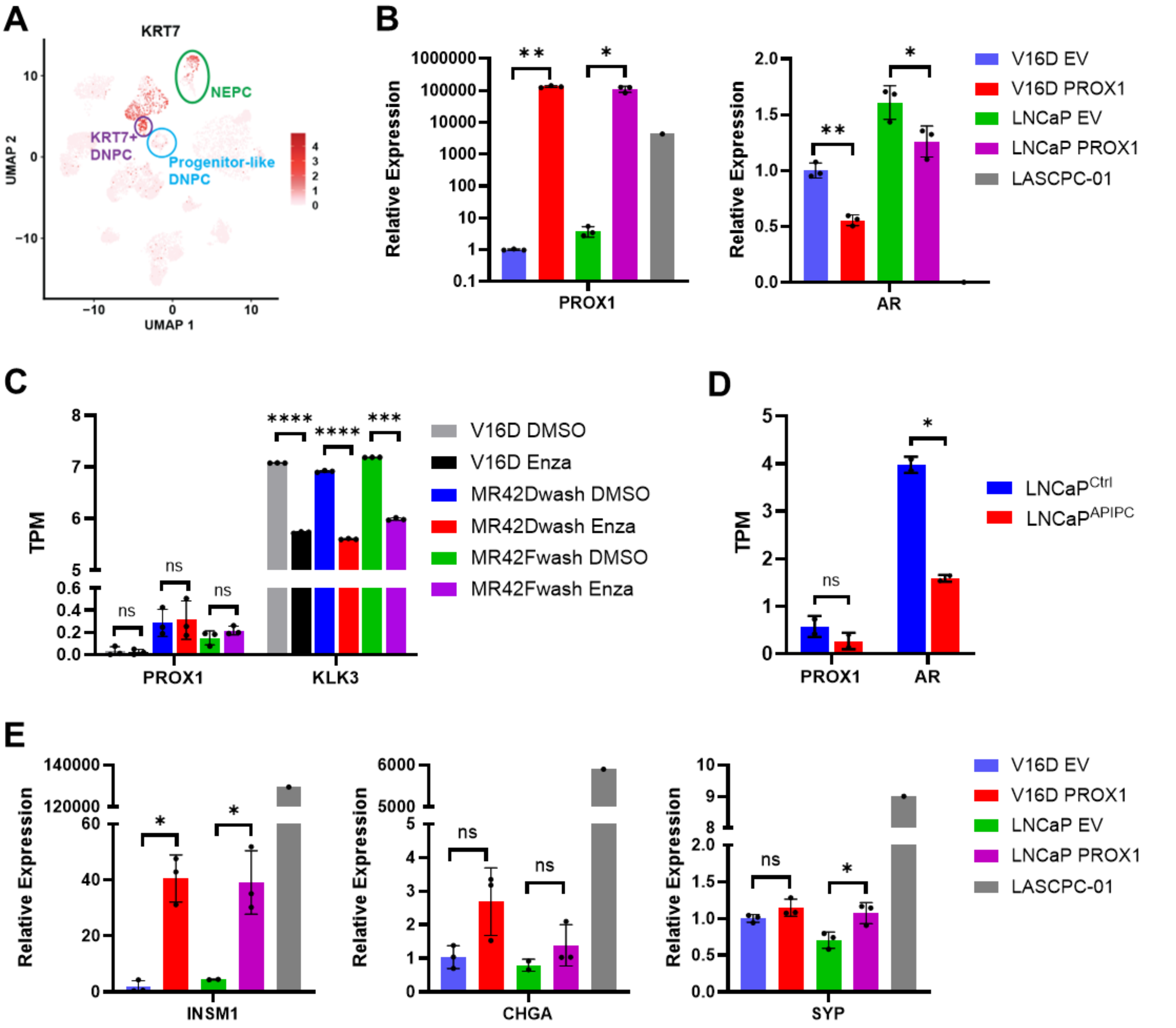

Supplemental Figure 4. PROX1 suppresses AR expression but is not sufficient to activate an NEPC program. **(A)** Feature plots of *KRT7* expression extracted from scRNA-seq meta-atlas published by Cheng et al. 2024 (Ref. 31) are shown. Populations of NEPC (green oval), *KRT7*+ DNPC (purple oval), and progenitor-like DNPC (blue circle) are marked according to the original publication. (**B**) Expression levels of indicated genes were measured by RT-qPCR in V16D or LNCaP cells transfected with empty vector (EV) or *PROX1* overexpression vector after 72 hours. LASCPC-01 serves as a positive control for *PROX1*. *Beta-actin* serves as a housekeeping control. (**C**) *PROX1* and *KLK3* expression were measured by RNA-seq from Kim et al. 2021 (Ref. 32) study using V16D, MR42D and MR42F (washout) with DMSO or enzalutamide (Enza) treatment. **(D)** *PROX1* and *AR* expression were measured by RNA-seq from Bluemn et al. 2017 (Ref. 3) using LNCaP^{APIPC} (*AR* KO) and its control line LNCaP$^{shAR/pATK}$ without Dox induction. (**E**) Expression levels of indicated NEPC markers were measured by RT-qPCR in V16D or LNCaP cells transfected with empty vector (EV) or *PROX1* overexpression vector from (B). LASCPC-01 serves as a positive control for NEPC markers. *Beta-actin* serves as a housekeeping control. For B-E, Data are reported as the mean ± SD. Statistical analysis was performed with a Student's t test with Welch's correction. **P*<0.05; ***P*<0.01; ****P*<0.001; *****P*<0.0001.

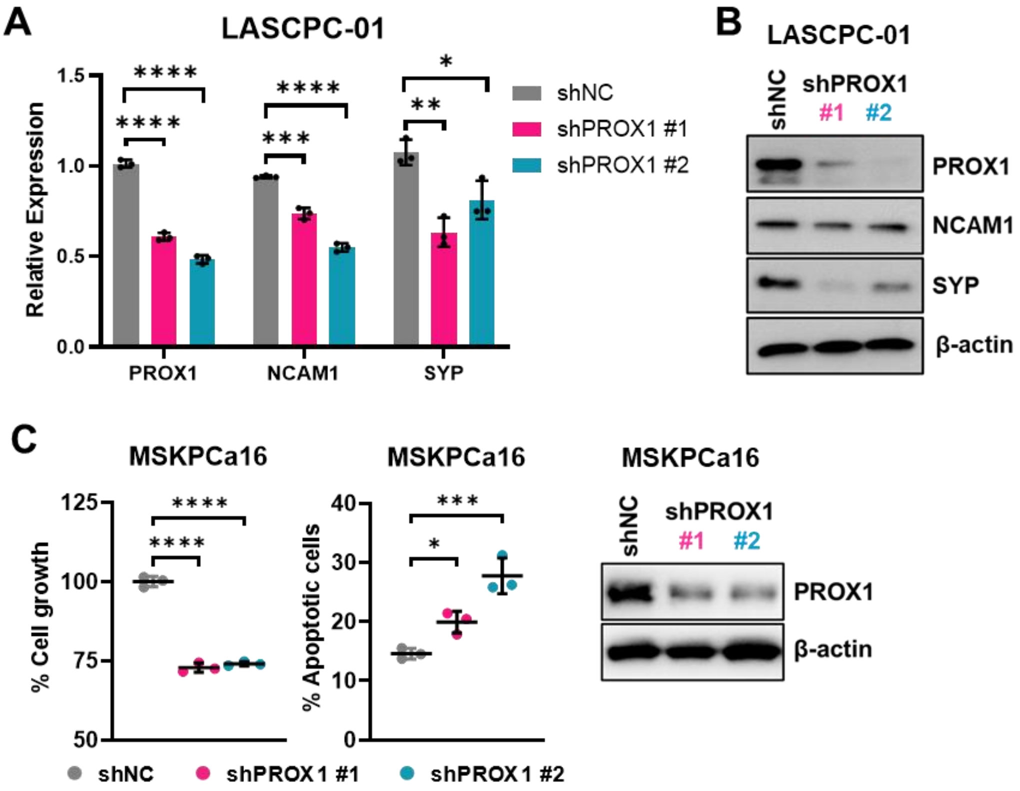

Supplemental Figure 5. ***PROX1*** **knockdown reduces NEPC differentiation markers and impairs growth of DNPC organoids. (A-B)** Expression of indicated genes was measured by RT-qPCR (A) or Western blots (B) in LASCPC-01 sh non-targeted control (shNC), shPROX1 #1 and #2 stable lines with doxycycline (1 μg/mL) treatment for total 8 days. **(C)** Cell growth and apoptosis was measured by Biospa Cytation 5 or Annexin V/Propidium iodide staining in MSKPCa16 shNC, shPROX1 #1 and #2 stable lines with doxycycline (1 μg/mL) treatment for total 10 days. PROX1 knockdown was measured by Western blots. For A and C, data are reported as the mean ± SD (N=3). Statistical analysis was performed by unpaired t test with Holm-Šídák method for multiple-comparison (A) and one-way ANOVA with Dunnett's multiple-comparison test (C). *P<0.05; **P<0.01; ***P<0.001; ****P<0.0001.

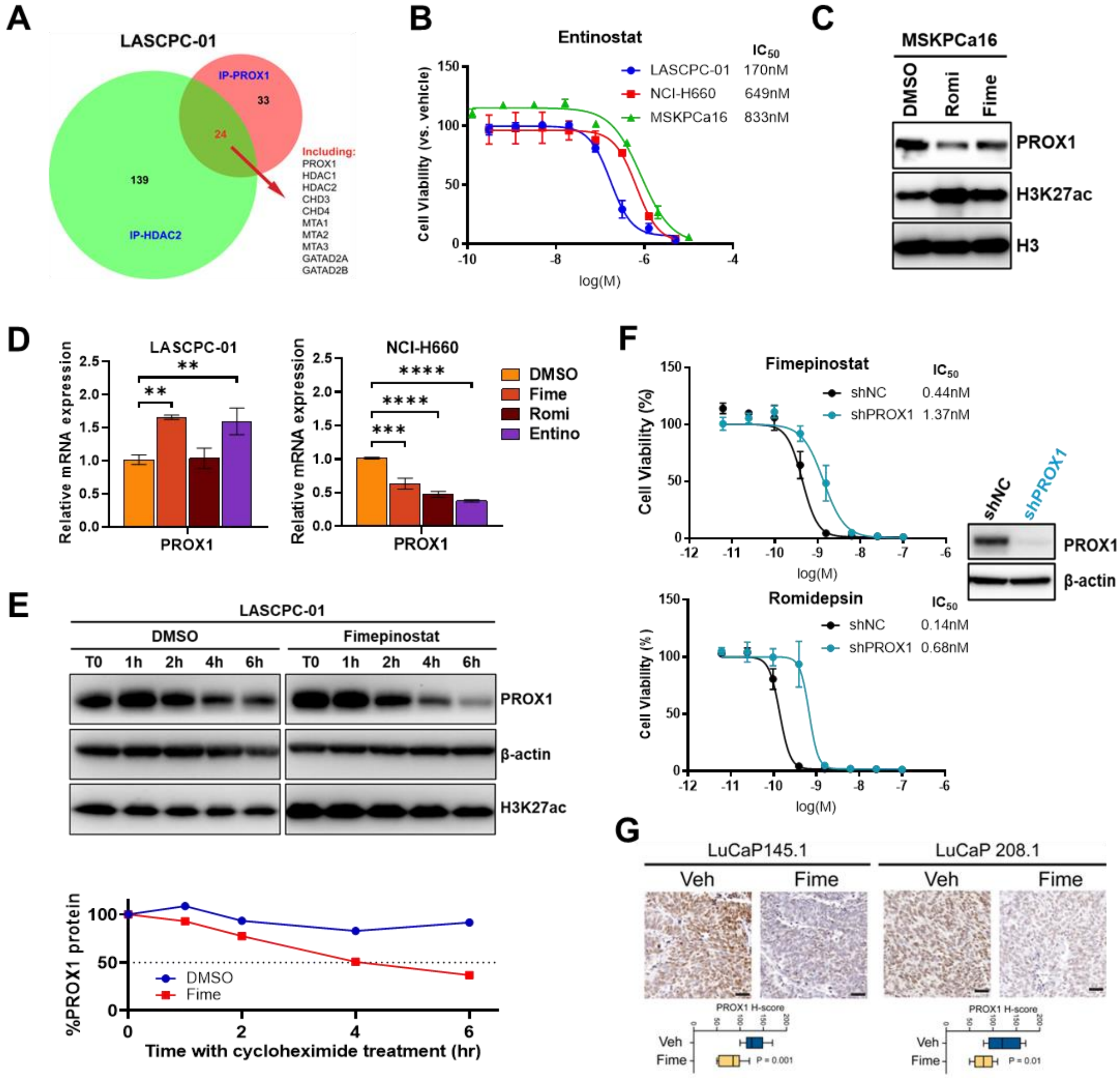

Supplemental Figure 6. Targeting PROX1-high models with HDAC inhibitors. (A) The number of interacting proteins for PROX1 or HDAC2 identified by immunoprecipitation and mass spectrometry in LASCPC-01 cells is shown in the Venn diagram. **(B)** Dose response (72 hrs) for Entinostat was measured by CellTiter-Glo (CTG) Luminescent Cell Viability Assays. Half-maximal inhibitory concentration (IC_{50}) was determined in the indicated cell lines. **(C)** DNPC MSKPCa16 organoids were treated with DMSO, Romidepsin (Romi, 0.8 nM) or Fimepinostat (Fime, 8 nM) for 48 hrs. Expression of the indicated proteins was measured by Western blots. **(D)** PROX1 expression with DMSO or HDAC inhibitor treatment in LASCPC-01 and NCI-H660 was measured by RT-qPCR. **(E)** PROX1 protein stability with DMSO or 5nM Fimepinostat treatment in LASCPC-01 cells was measured after adding cycloheximide (10 μg/mL). **(F)** Dose response (72 hrs) for Fimepinostat or Romidepsin in NCI-H660 with *PROX1* shRNA or non-targeted control (NC) shRNA was measured by CTG assays. IC_{50} values are shown, and PROX1 knockdown was confirmed by Western blots. **(G)** PROX1 IHC and staining scores was shown for LuCaP 145.1 and LuCaP 208.1 PDXs that were treated with vehicle or Fimepinostat *in vivo* in Zhang et al. 2023 (Ref. 34). For D, data are reported as the mean ± SD (N=3). Statistical analysis was performed by one-way ANOVA with Dunnett's multiple-comparison test. ***P*<0.01; ****P*<0.001; *****P*<0.0001.

Supplemental Methods

Immunoprecipitation

Cells or PDX tumor pieces were lysed in IP Lysis Buffer (Thermo Scientific, cat# 87787) with protease inhibitor (Thermo Scientific, cat# A32955), then the protein concentration was measured by Rapid Gold BCA Protein Assay Kit (Thermo Scientific, cat# A53225). Immunoprecipitation was performed by using 2mg total protein with 2 µg rabbit IgG (Millipore, cat# 12-370), anti-PROX1 (Proteintech, cat#11067-2-AP) or anti-HDAC2 (Abam, cat# ab219053) incubated overnight at 4°C, then Dynabeads Protein G (Invitrogen cat# 10004D) was used to pull-down the complex following by washing with IP Lysis Buffer four times and cold PBS once. Beads were boiled with SDS loading buffer for 10 minutes for running Western Blots assays or beads were saved at -80°C for Mass Spectrometry assays.

Mass Spectrometry

The beads samples were submitted to Proteomics Resource Facility at University of Michigan for mass spectrometry assays on a fee-for-service basis. Briefly, the beads were resuspended in 50 mL of 0.1 M ammonium bicarbonate buffer (pH 8). Cysteines were reduced by adding 50 mL of 10 mM DTT and incubating at 45°C for 30 min. Samples were cooled to room temperature and alkylation of cysteines was achieved by incubating with 65 mM 2-Chloroacetamide, under darkness, for 30 min at room temperature. An overnight (~16 hours) digestion with 1 µg trypsin was carried out at 37°C with constant shaking in a Thermomixer. Digestion was stopped by acidification and peptides were desalted using SepPak C18 cartridges using the manufacturer's protocol (Waters). Samples were completely dried using vacufuge. Resulting peptides were dissolved in 8 mL of 0.1% formic acid/2% acetonitrile solution and 2 mL of the peptide solution were resolved on a nano-capillary reverse phase column (Acclaim PepMap C18, 2 micron, 50 cm, ThermoScientific) using a 0.1% formic acid/2% acetonitrile (Buffer A) and 0.1% formic acid/95% acetonitrile (Buffer B) gradient at 300 nL/min over a period of 90 min (2-25% buffer B in 45 min, 25-40% in 5 min, 40-90% in 5 min followed by holding at 90% buffer B for 5 min and equilibration with

Buffer A for 30 min). Eluent was directly introduced into Orbitrap Fusion tribrid mass spectrometer (Thermo Scientific, San Jose CA) using an EasySpray source. MS1 scans were acquired at 120K resolution (AGC target=2x10^5; max IT=100 ms). Data-dependent High-energy C-trap dissociation MS/MS spectra were acquired using Top speed method (3 seconds) following each MS1 scan (NCE ~32%; AGC target 5x10^4; max IT 50 ms, 15K resolution). Proteins were identified by searching the MS/MS data against Human protein database (20291 entries; reviewed; downloaded on 12/13/2021) using Proteome Discoverer (v2.4, Thermo Scientific). Search parameters included MS1 mass tolerance of 10 ppm and fragment tolerance of 0.2 Da; two missed cleavages were allowed; carbamidimethylation of cysteine was considered fixed modification and oxidation of methionine, deamidation of asparagine and glutamine were considered as potential modifications. False discovery rate (FDR) was determined using Percolator and proteins/peptides with an FDR of ≤1% were retained for further analysis.
