# Supplementary material for: PROX1 is an early driver of lineage plasticity in prostate cancer
Source: J Clin Invest. 2025 Jun 2;135(11):e187490. doi: 10.1172/JCI187490 (PMC12126232; doi:10.1172/JCI187490)

Figure 2B

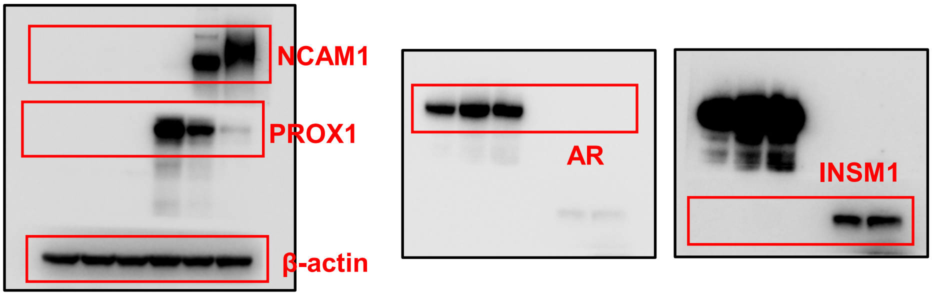

Suppl Figure 2C

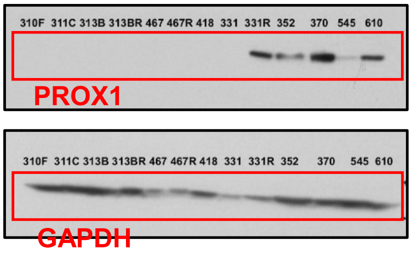

Figure 3D

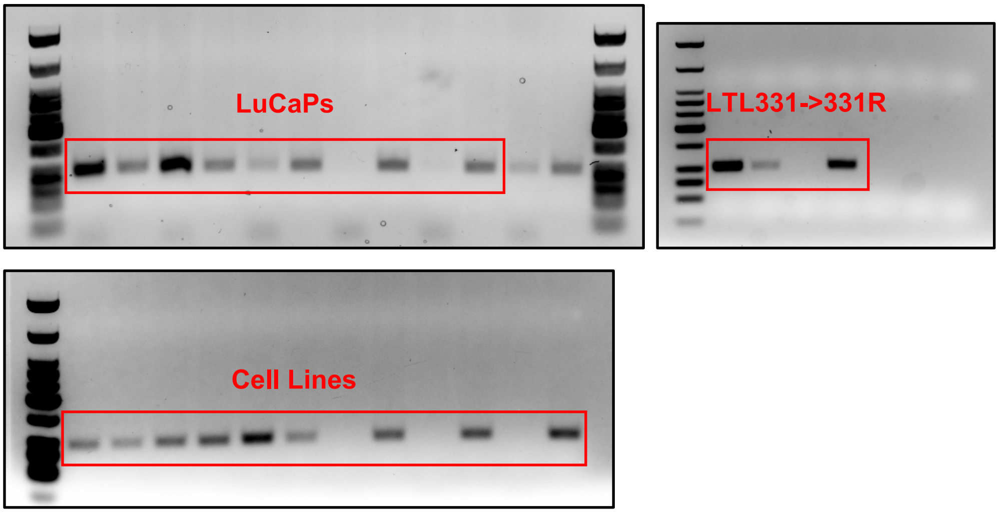

Figure 4F

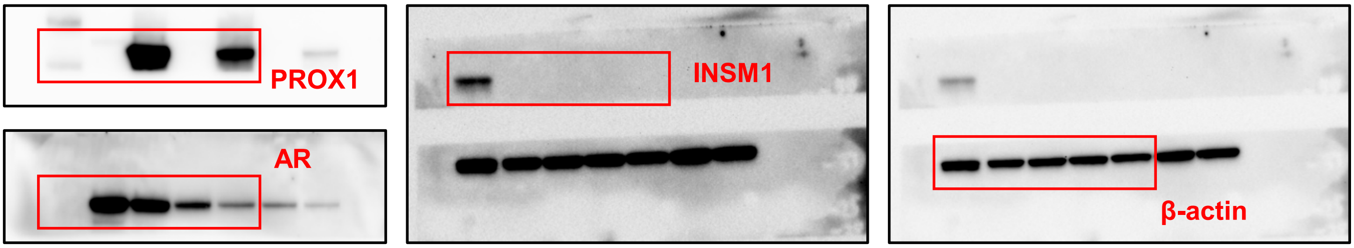

Figure 5A

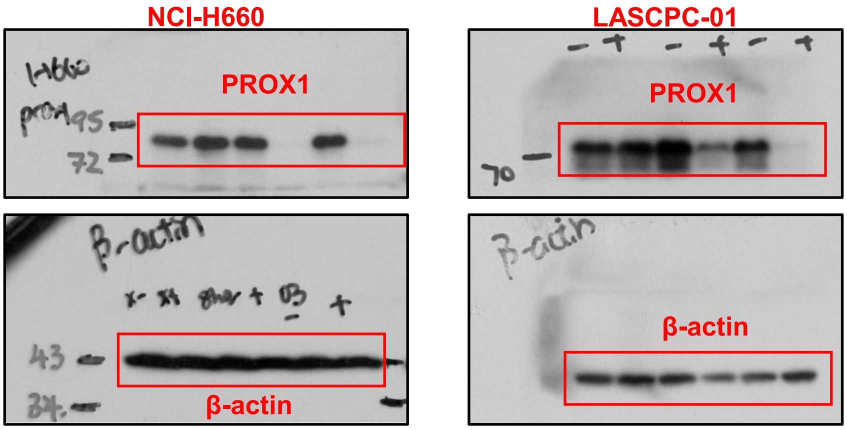

Figure 5D

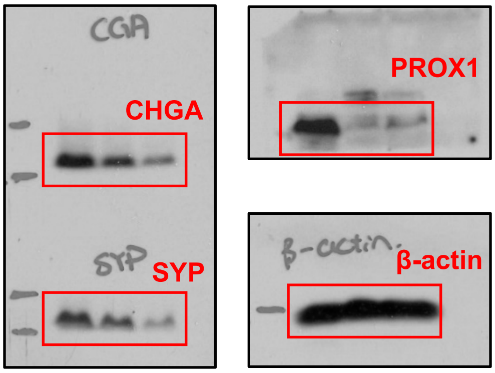

Suppl Figure 5B

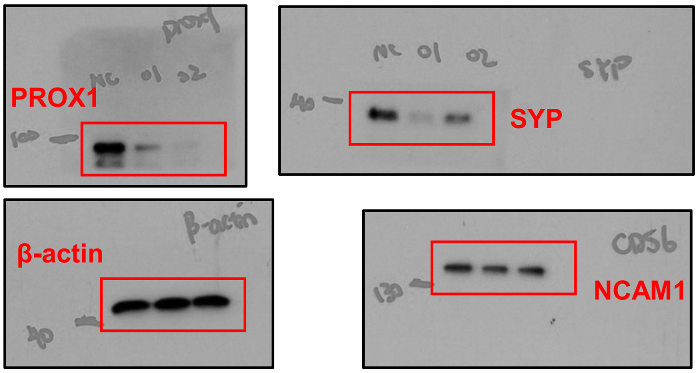

Suppl Figure 5C

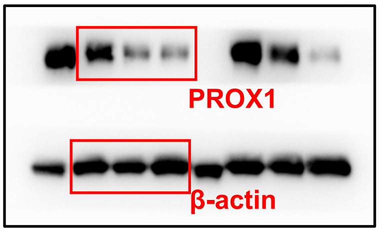

Figure 6A

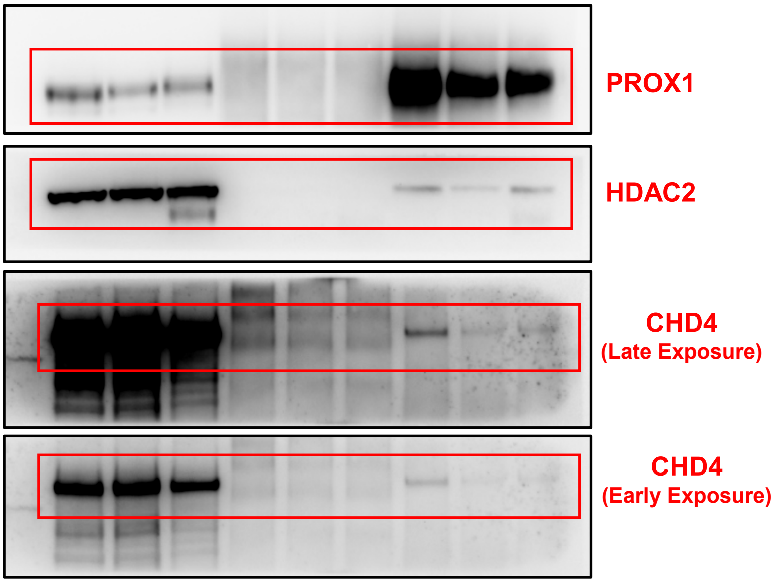

Figure 6C

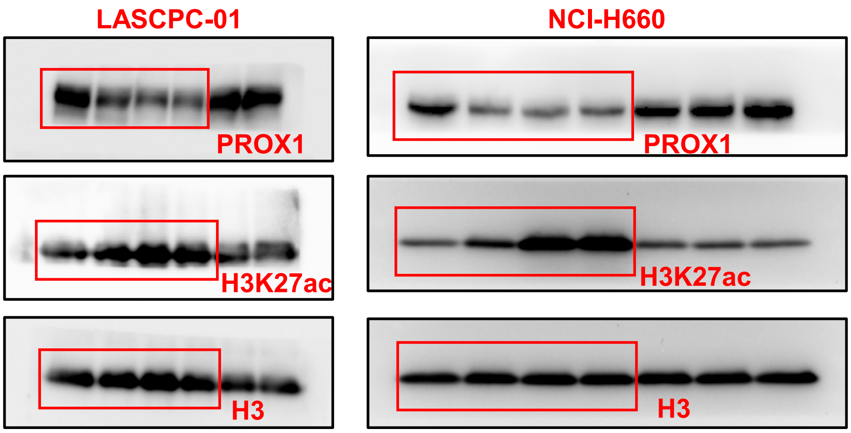

Figure 6F

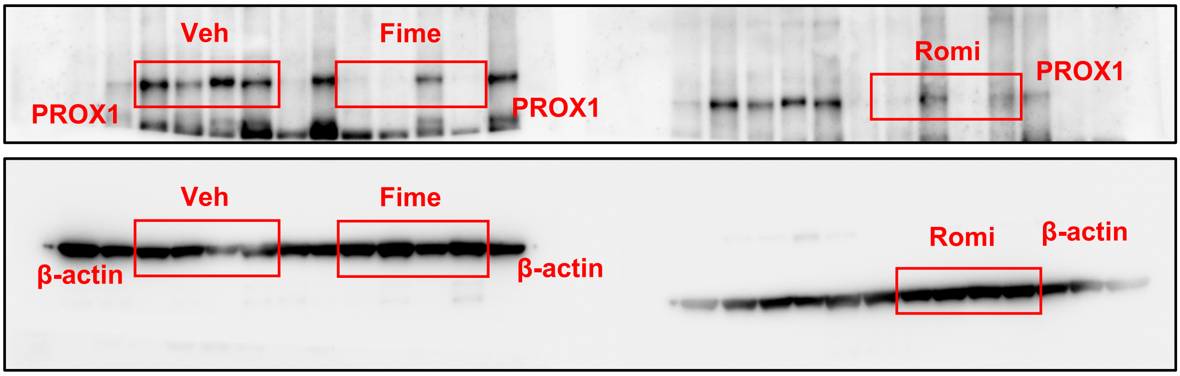

Suppl Figure 6C

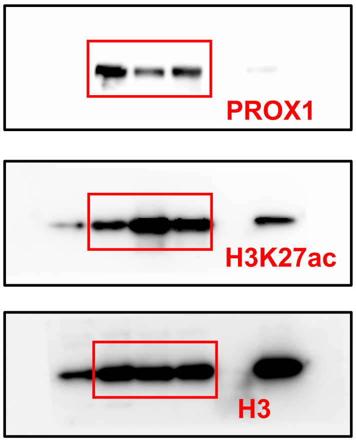

Suppl Figure 6E

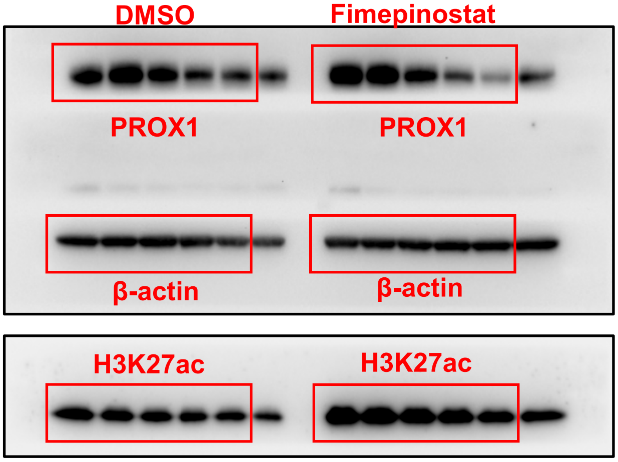

Suppl Figure 6F

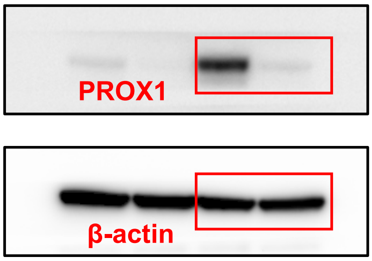

Supplement: Unedited blot and gel images [file jci-135-187490-s271.pdf]
